# Metabolic engineering of a tyrosine-overproducing yeast platform using targeted metabolomics

**DOI:** 10.1186/s12934-015-0252-2

**Published:** 2015-05-28

**Authors:** Nicholas D. Gold, Christopher M. Gowen, Francois-Xavier Lussier, Sarat C. Cautha, Radhakrishnan Mahadevan, Vincent J. J. Martin

**Affiliations:** Department of Biology and Centre for Structural and Functional Genomics, Concordia University, 7141 Sherbrooke West, Montreal, QC H4B 1R6 Canada; Department of Chemical Engineering and Applied Chemistry, University of Toronto, 200 College Street, Toronto, ON M5S 3E5 Canada; Institute of Biomaterials and Biomedical Engineering, University of Toronto, 164 College Street, Toronto, ON M5S 3G9 Canada

**Keywords:** L-tyrosine, *Saccharomyces cerevisiae*, Metabolic engineering, Targeted metabolomics, Glucose-6-phosphate dehydrogenase, Pyruvate kinase, Prephenate dehydrogenase, Cyclohexadienyl dehydrogenase, Phenylpyruvate decarboxylase, Aromatic amino acids

## Abstract

**Background:**

L-tyrosine is a common precursor for a wide range of valuable secondary metabolites, including benzylisoquinoline alkaloids (BIAs) and many polyketides. An industrially tractable yeast strain optimized for production of L-tyrosine could serve as a platform for the development of BIA and polyketide cell factories. This study applied a targeted metabolomics approach to evaluate metabolic engineering strategies to increase the availability of intracellular L-tyrosine in the yeast *Saccharomyces cerevisiae* CEN.PK. Our engineering strategies combined localized pathway engineering with global engineering of central metabolism, facilitated by genome-scale steady-state modelling.

**Results:**

Addition of a tyrosine feedback resistant version of 3-deoxy-D-arabino-heptulosonate-7-phosphate synthase Aro4 from *S. cerevisiae* was combined with overexpression of either a tyrosine feedback resistant yeast chorismate mutase Aro7, the native pentafunctional arom protein Aro1, native prephenate dehydrogenase Tyr1 or cyclohexadienyl dehydrogenase TyrC from *Zymomonas mobilis*. Loss of aromatic carbon was limited by eliminating phenylpyruvate decarboxylase Aro10. The *TAL* gene from *Rhodobacter sphaeroides* was used to produce coumarate as a simple test case of a heterologous by-product of tyrosine. Additionally, multiple strategies for engineering global metabolism to promote tyrosine production were evaluated using metabolic modelling. The T21E mutant of pyruvate kinase Cdc19 was hypothesized to slow the conversion of phosphoenolpyruvate to pyruvate and accumulate the former as precursor to the shikimate pathway. The *ZWF1* gene coding for glucose-6-phosphate dehydrogenase was deleted to create an NADPH deficiency designed to force the cell to couple its growth to tyrosine production via overexpressed NADP^+^-dependent prephenate dehydrogenase Tyr1. Our engineered Zwf1^−^ strain expressing *TYRC ARO4*^*FBR*^ and grown in the presence of methionine achieved an intracellular L-tyrosine accumulation up to 520 μmol/g DCW or 192 mM in the cytosol, but sustained flux through this pathway was found to depend on the complete elimination of feedback inhibition and degradation pathways.

**Conclusions:**

Our targeted metabolomics approach confirmed a likely regulatory site at DAHP synthase and identified another possible cofactor limitation at prephenate dehydrogenase. Additionally, the genome-scale metabolic model identified design strategies that have the potential to improve availability of erythrose 4-phosphate for DAHP synthase and cofactor availability for prephenate dehydrogenase. We evaluated these strategies and provide recommendations for further improvement of aromatic amino acid biosynthesis in *S. cerevisiae*.

**Electronic supplementary material:**

The online version of this article (doi:10.1186/s12934-015-0252-2) contains supplementary material, which is available to authorized users.

## Background

*Saccharomyces cerevisiae* is the host of choice for production of high value plant-specific secondary metabolites with numerous pharmaceutical, industrial, and nutritional applications [1–5]. Commercialization of this technology depends on significant improvements in product yield, titre, and productivity. Although targeted optimization of heterologous pathways will be vital to maximize product yields, a more general strategy with some promise is to develop platform strains that are engineered for the production of common plant metabolite precursors. The aromatic amino acid L-tyrosine is a key precursor in the biosynthesis of both polyketides and benzylisoquinoline alkaloids [6], making it a useful target for metabolic engineering in yeast.

**Fig. 1:**
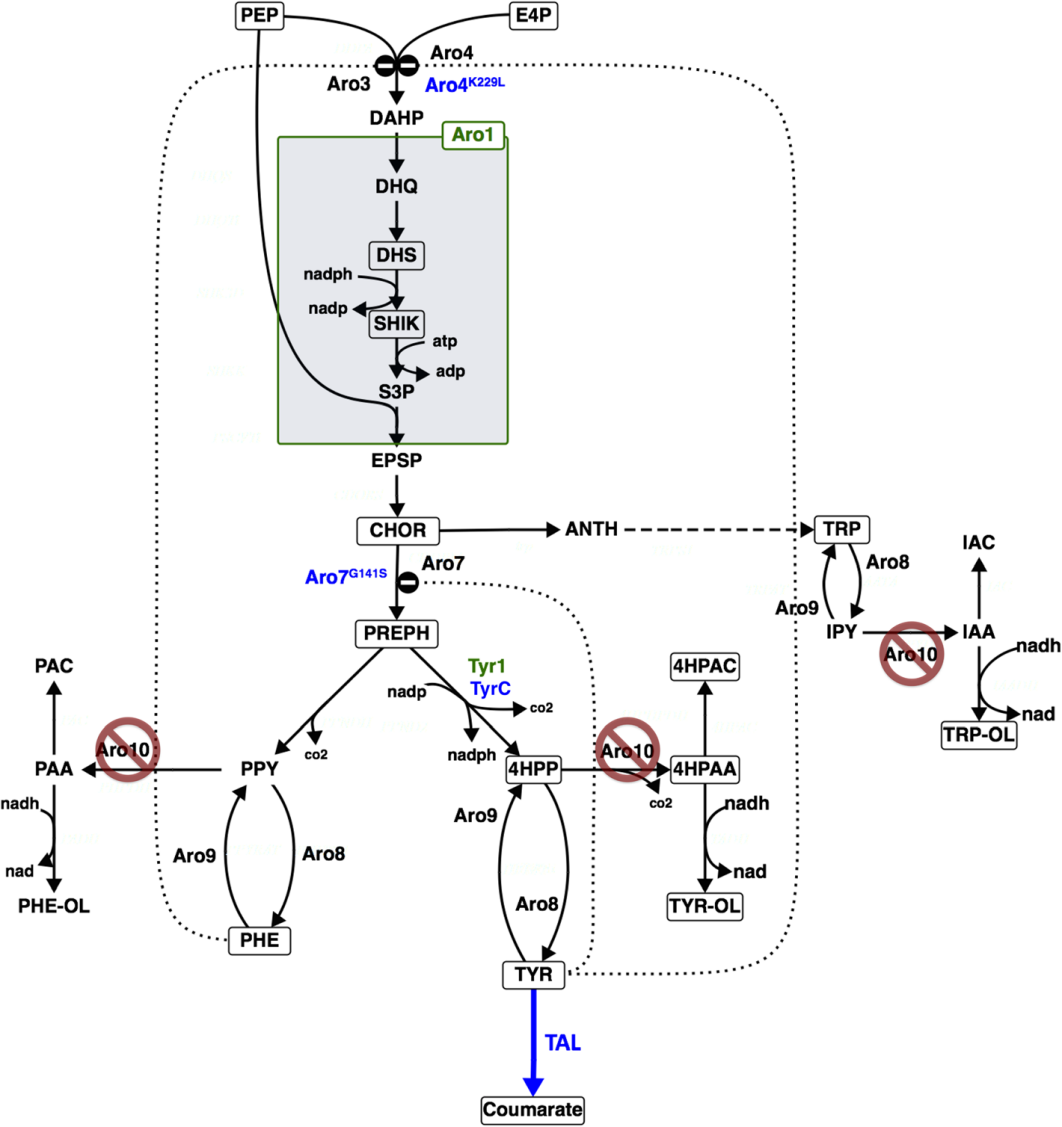
Aromatic amino acid biosynthesis and degradation pathways in *S. cerevisiae*. The native aromatic amino acid biosynthesis and degradation pathways are indicated with solid *black* arrows. Overexpression of the non-native or engineered enzymes is indicated using *blue* font, including tyrosine ammonia lyase (TAL) from *R. sphaeroides* [19] and the NAD^+^-dependent prephenate dehydrogenase (TyrC) from *Z. mobilis*, the feedback-resistant DAHP synthase Aro4^K229L^, and the feedback-resistant chorismate mutase Aro7^G141S^. Native genes that are overexpressed in this study are shown using a *green* font, while knockout of the first step in the aromatic amino acid degradation pathway, Aro10, is indicated by a ‘prohibited’ symbol. *Dotted lines* indicate allosteric inhibition by phenylalanine of Aro3 and by tyrosine of Aro4 and Aro7. Boxed metabolites were measured in this study. Metabolite abbreviations: PEP, phosphoenolpyruvate; E4P, erythrose-4-phosphate; DAHP, 3-deoxy-D-arabinoheptulosonate-7-phosphate; DHQ, 3-dehydroquinate; DHS, dehydroshikimate; SHIK, shikimate; S3P, shikimate-3-phosphate; EPSP, 5-enolpyruvyl-shikimate-3-phosphate; CHOR, chorismate; ANTH, anthranilate; TRP, L-tryptophan; IPY, indole pyruvate; IAA, indole acetaldehyde; IAC, indole acetate; TRP-OL, tryptophol; PREPH, prephenate; PPY, phenylpyruvate; PHE, L-phenylalanine; PAA, phenylacetaldehyde; PAC, phenylacetate; PHE-OL, phenylethanol; TYR, L-tyrosine; COU, coumarate; 4HPP, 4-hydroxyphenylpyruvate; 4HPAA, 4-hydroxyphenylacetaldehyde; 4HPAC, 4-hydroxyphenylacetate; TYR-OL, tyrosol

Synthesis of aromatic amino acids in yeast proceeds via the shikimate pathway, which consists of seven enzymatic reactions leading to the generation of chorismate, the common precursor to all three aromatic amino acids (Fig. 1) [7]. The first committed step of the shikimate pathway is the condensation of phosphoenolpyruvate (PEP) and erythrose 4-phosphate (E4P) to form 3-deoxy-D-arabino-heptulosonate-7-phosphate (DAHP). In yeast this reaction is catalyzed by one of two DAHP synthase (EC 2.5.1.54) isozymes, Aro3 and Aro4, which are allosterically inhibited by phenylalanine and tyrosine, respectively [8]. DAHP is then consumed by Aro1, a pentafunctional enzyme that catalyzes five reactions including shikimate synthesis from dehydroshikimate (DHS) [9]. The last conversion step to chorismate is carried out by chorismate synthase Aro2 (EC 4.2.3.5). Carbon is diverted away from the tryptophan biosynthesis branch by the activity of chorismate mutase Aro7 (EC 5.4.99.5), which catalyzes the conversion of chorismate to prephenate, the last precursor common to both phenylalanine and tyrosine. Aro7 is allosterically inhibited by tyrosine and activated by tryptophan [10]. Prephenate dehydrogenase Tyr1 (EC 1.3.1.12) catalyzes the conversion of prephenate to the α-keto acid 4-hydroxyphenylpyruvate (4HPP). Finally, both Aro8 (EC 2.6.1.57) and Aro9 (EC 2.6.1.58) can reversibly transaminate 4HPP to L-tyrosine.

Previous approaches to engineering aromatic amino acid overproduction in yeast have generally focused on both deregulation of the key checkpoints in tyrosine biosynthesis and the removal of degradation pathways [11]. The essential regulatory modification is the removal of feedback inhibition of DAHP synthase. In previous work, knocking out *ARO3* and *ARO4* and overexpressing feedback-resistant mutants of *ARO4* and *ARO7* resulted in a 5.5-fold increase in intracellular tyrosine and a 200-fold increase in extracellular aromatic compounds relative to a reference strain in chemostat growth, corresponding to a 4.5-fold flux increase through the aromatic amino acid biosynthesis pathway [11]. Additionally, while many prephenate dehydrogenases are allosterically inhibited by tyrosine or 4HPP [12], *TYR1* is only known to be regulated transcriptionally by phenylalanine [13], although *TYR1* has not been previously targeted for metabolic engineering purposes.

Tyrosine degradation proceeds via the Ehrlich pathway, in which 4HPP is decarboxylated by phenylpyruvate decarboxylase Aro10 or by pyruvate decarboxylases [14] (Fig. 1). The resulting aldehyde can then either be oxidized to 4-hydroxyphenylethanol (tyrosol) or reduced to 4-hydroxyphenylacetate (4HPAC). Koopman et al. showed reduced loss of tyrosine to Ehrlich pathway by-product formation by eliminating Aro10, as well as pyruvate decarboxylases Pdc5 and Pdc6 [15].

This study systematically combines these localized pathway engineering approaches with global engineering of central metabolism, facilitated by steady-state modelling. A genome-scale model of yeast metabolism, iMM904 [16] was used in the steady-state strain design algorithms, OptKnock [17] and GDLS [18] to identify genes to overexpress or delete to enhance the tyrosine yield of *S. cerevisiae*. A targeted metabolomics approach was employed to query the effects of each of the genetic variations applied. In all, nineteen metabolites from glucose to coumarate, via the aromatic amino acid production pathway, were monitored over time. The contribution of tyrosine pools toward potential downstream use was evaluated by catalyzing the conversion of tyrosine to coumarate using tyrosine ammonia lyase (TAL; EC 4.3.1.23) from *Rhodobacter sphaeroides* [19]. This enzyme represents the first step in the production of many polyketides, including naringenin and the prenylated flavonoid xanthohumol from hops (*Humulus lupus*) [19].

## Results

**Fig. 2:**
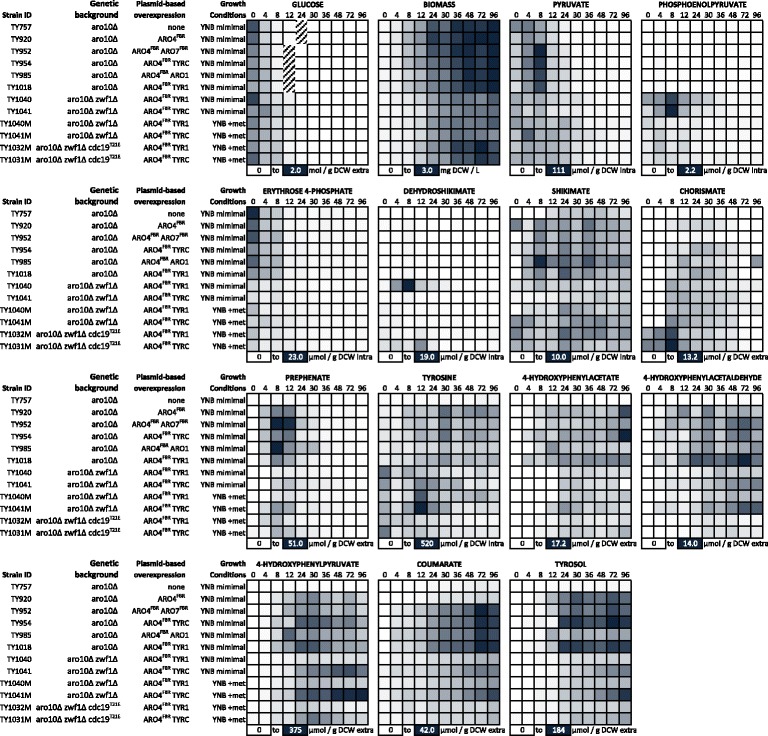
Targeted metabolite analysis of *S. cerevisiae* strains used in this study. Complete phenotypic descriptions of the strains are given in Table 1. Metabolite levels are shown in specific concentrations per g DCW. Low and high ends of concentration ranges per metabolite represented by *white* and *black*, respectively. Abbreviations: PEP, phosphoenolpyruvate; E4P, erythrose 4-phosphate; DHS, dehydroshikimate; PPH/PPY, prephenate/phenylpyruvate measured as a mixed peak by HPLC-PDA; 4HPP, 4-hydroxyphenylpyruvate; 4HPAA, 4-hydroxyphenylacetaldehyde; 4HPAC, 4-hydroxyphenylacetate. Intra and extra denote intra-cellular and extra-cellular metabolites, respectively

In this study, a targeted metabolomics approach was employed to systematically examine the impacts of multiple metabolic engineering strategies for the production of tyrosine in *S. cerevisiae*. Specific concentrations of several components of the aromatic amino acid biosynthesis pathway over the fermentation time course are shown in Fig. 2, and additional metabolite concentrations are included as additional information (Additional file 1: Figure S1, Additional file 2: Figure S2, Additional file 3: Figure S3, Additional file 4: Figure S4, Additional file 5: Figure S5, Additional file 6: Figure S6). These concentration data were also used to estimate the changes in Gibbs free energy of reaction across the pathway (Additional file 7: Figure S7). A minimally engineered reference strain (TY920; Table 1 and Additional file 8: Table S1) harboured the following modifications compared to wild-type: (i) the committed first step into the shikimate pathway was deregulated by overexpression of the K229L tyrosine feedback-resistant mutant *ARO4*^*FBR*^ allele in an *ARO3 ARO4* haploid background; (ii) the *ARO10* gene was deleted to reduce the loss of aromatic amino acid carbon to the Ehrlich pathway; and (iii) the *TAL* gene from *R. sphaeroides* was overexpressed to enable transformation of tyrosine into coumarate as a test of the strain’s capacity to host heterologous pathways deriving from tyrosine. This strain represents a basic set of modifications found to be important to tyrosine or phenylalanine production by previous studies [15].

**Table 1:** *Saccharomyces cerevisiae* strains tested in this study

Strain name	Phenotype of host	Genes added on plasmids
TY757	Aro10^−^ Zwf1^+^ Cdc19^+^	*TAL*
TY920	Aro10^−^ Zwf1^+^ Cdc19^+^	*TAL ARO4* ^*FBR*^
TY985	Aro10^−^ Zwf1^+^ Cdc19^+^	*TAL ARO4* ^*FBR*^ *ARO1*
TY952	Aro10^−^ Zwf1^+^ Cdc19^+^	*TAL ARO4* ^*FBR*^ *ARO7* ^*FBR*^
TY954	Aro10^−^ Zwf1^+^ Cdc19^+^	*TAL ARO4* ^*FBR*^ *TYRC*
TY1018	Aro10^−^ Zwf1^+^ Cdc19^+^	*TAL ARO4* ^*FBR*^ *TYR1*
TY1041	Aro10^−^ Zwf1^−^ Cdc19^+^	*TAL ARO4* ^*FBR*^ *TYRC*
TY1040	Aro10^−^ Zwf1^−^ Cdc19^+^	*TAL ARO4* ^*FBR*^ *TYR1*
TY1031	Aro10^−^ Zwf1^−^ Cdc19^low^	*TAL ARO4* ^*FBR*^ *TYRC*
TY1032	Aro10^−^ Zwf1^−^ Cdc19^low^	*TAL ARO4* ^*FBR*^ *TYR1*

### Aromatic amino acid pathway engineering

**Fig. 3:**
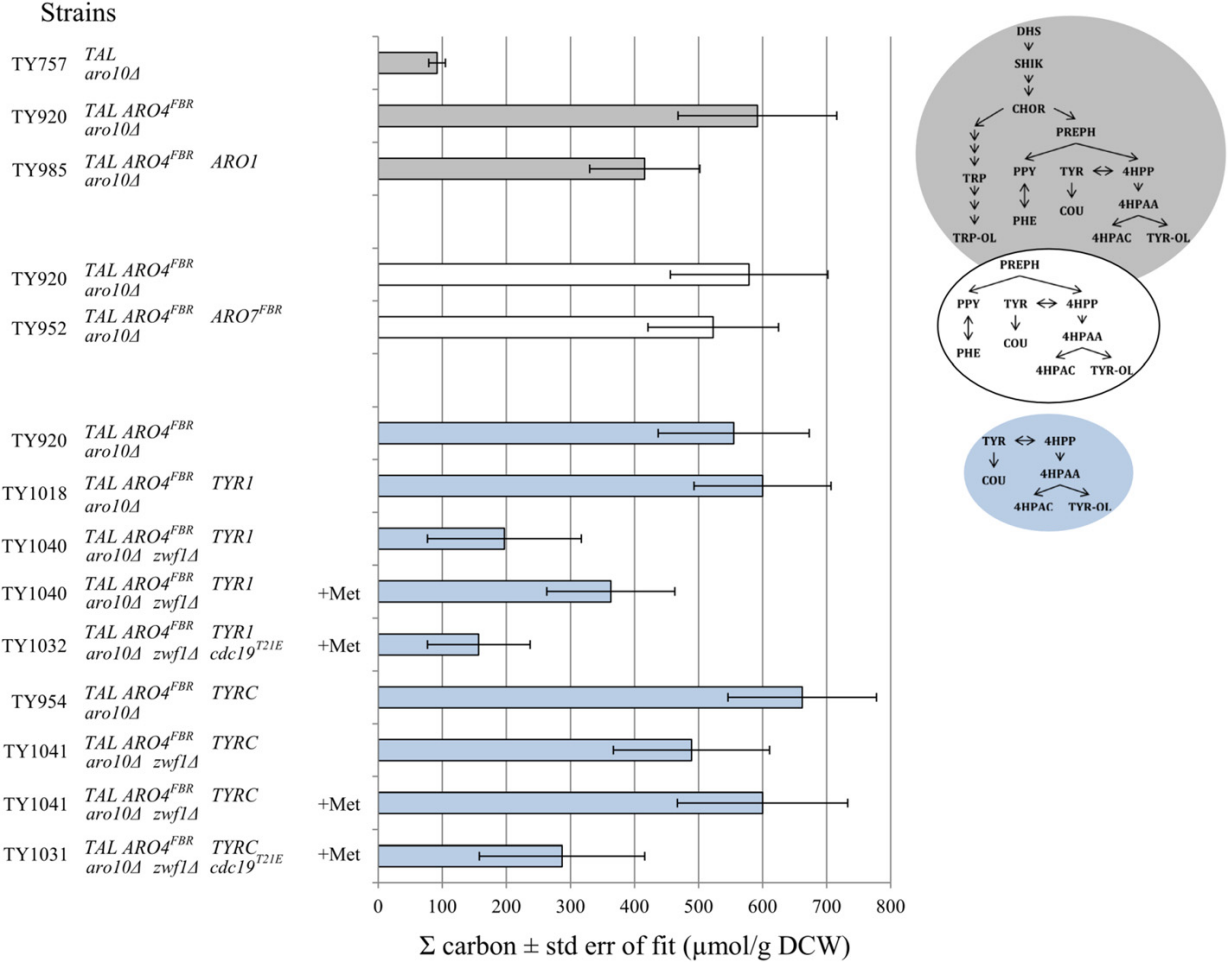
Total specific carbon measured for metabolites affected by engineering of localized pathway and global metabolism. Estimated using a single 2-parameter exponential rise to maximum curve fit in SigmaPlot11.0 and values taken at 48 h. Referring always to strain TY920, strains TY757 and TY985 were evaluated downstream of DHS inclusively, strain TY952 downstream of prephenate/phenylpyruvate inclusively, and *TYR1* and *TYRC* strains, grown with or without methionine, downstream of 4HPP inclusively

Relative to the control strain TY757, overexpression of *ARO4*^*FBR*^ in TY920 improved the coumarate yield by more than two-fold (Fig. 2) and total specific carbon measured downstream of the condensation of PEP and E4P by greater than six-fold (Fig. 3) without disturbing growth or overflow metabolism (Tables 2 and 3). This represents a carbon increase of about 0.5 mmol/g DCW to the aromatic amino acid pathway. Levels of PEP and E4P did not change, but every metabolite measured downstream of DHS inclusively showed an increase (Fig. 2 and Additional file 1: Figure S1-A). Thus, as demonstrated previously [11, 15], adding a deregulated DAHP synthase had a clear carbon benefit to the shikimate and aromatic amino acid pathways.

**Table 2:** Growth characteristics and glucose uptake

Strain	YNB + Gluc	μ_MAX_	Y_X/S_	Glucose uptake
			(g DCW/g glucose)	(mmol/g DCW/h)
TY757		0.168 ± 0.021	0.0041 ± 0.0003	94.2 ± 14.0
TY920		0.162 ± 0.035	0.0040 ± 0.0004	88.9 ± 38.9
TY952		0.161 ± 0.030	0.0038 ± 0.0003	94.0 ± 21.1
TY985		0.163 ± 0.021	0.0038 ± 0.0004	121.3 ± 30.9
TY1018		0.170 ± 0.024	0.0038 ± 0.0005	120.6 ± 40.0
TY954		0.167 ± 0.017	0.0037 ± 0.0010	105.3 ± 23.5
TY1040		0.061 ± 0.019	0.0024 ± 0.0004	55.6 ± 18.2
TY1040	w/ Met	0.088 ± 0.008	0.0025 ± 0.0005	60.4 ± 18.9
TY1032	w/ Met	0.059 ± 0.004	0.0035 ± 0.0005	61.6 ± 15.5
TY1041		0.072 ± 0.006	0.0024 ± 0.0001	100.2 ± 34.8
TY1041	w/ Met	0.089 ± 0.018	0.0022 ± 0.0002	70.7 ± 23.1
TY1031	w/ Met	0.080 ± 0.011	0.0033 ± 0.0002	57.8 ± 14.6

**Table 3:** Overflow metabolism of acetate

Strain	YNB + Gluc	Acetate production	Acetate max @ time	Acetate re-uptake
TY757		0.977 ± 0.149	12.9 ± 2.2 @ 12 h	0.672 ± 0.125
TY920		0.918 ± 0.142	11.0 ± 1.6 @ 12 h	0.693 ± 0.209
TY952		1.261 ± 0.075	14.4 ± 0.4 @ 12 h	0.698 ± 0.019
TY985		1.742 ± 0.072	23.2 ± 1.3 @ 12 h	1.433 ± 0.150
TY1018		1.750 ± 0.118	20.2 ± 0.7 @ 12 h	1.018 ± 0.061
TY954		1.517 ± 0.270	12.2 ± 2.6 @ 8 h	0.417 ± 0.012
TY1040		0.754 ± 0.320	9.5 ± 0.3 @ 24 h	0.377 ± 0.145
TY1040	w/ Met	1.091 ± 0.111	10.7 ± 1.3 @ 12 h	0.276 ± 0.073
TY1032	w/ Met	2.239 ± 0.664	19.0 ± 6.2 @ 8 h	0.679 ± 0.154
TY1041		1.994 ± 0.694	15.1 ± 1.5 @ 12 h	0.224 ± 0.027
TY1041	w/ Met	1.578 ± 0.389	12.8 ± 0.9 @ 24 h	0.351 ± 0.215
TY1031	w/ Met	1.551 ± 0.209	13.5 ± 1.6 @ 8 h	0.403 ± 0.071

All uptake/production rates in mmol/g DCW/h. Maximum detected values in mmol/g DCW

To improve on the output of strain TY920 we tested the relative impact of three separate strategies, each one overexpressing a second gene in addition to *ARO4*^*FBR*^ in the Aro10^−^ host (Table 1): the native *ARO1* gene in strain TY985 to increase pull on the product of Aro4^K229L^; the G141S tyrosine feedback-resistant mutant chorismate mutase *ARO7*^*FBR*^ allele in strain TY952 to deregulate control over the branch point between tryptophan and tyrosine/phenylalanine [11]; and either the native prephenate dehydrogenase *TYR1* in strain TY1018 or cyclohexadienyl dehydrogenase *TYRC* (EC 1.3.1.79) from the bacterium *Zymomonas mobilis,* which is known to be feedback-insensitive to tyrosine [12], in strain TY954 to increase the conversion of prephenate to 4HPP (Additional file 1: Figure S1-B to D). Maximum specific growth rate, growth yield, and glucose uptake rate were not affected by overexpression of *ARO1*, *ARO7*^*FBR*^, *TYR1* or *TYRC* in addition to *ARO4*^*FBR*^ (Table 2). Interestingly, in all strains, *ΔG*_*γ*_ of the reactions DAHP synthase catalyzed by Aro4 and 3DHQ synthase catalyzed by the first step of Aro1 (Additional file 7: Figure S7) was estimated to be greatest in magnitude. Although in this estimation, the concentrations of DAHP and 3DHQ were not measured and assumed to be 1 mM, this finding is consistent with the hypothesis that these reactions are maintained furthest from equilibrium and are therefore most likely to be actively regulated [20].

In order to evaluate the capacity of the engineered strains for industrial polyketide or alkaloid production, we overexpressed the *TAL* gene in all strains [19]. In the absence of *TAL*, coumarate was never detected. While *S. cerevisiae* W303 is known to consume coumarate via the activity of phenylacrylic acid decarboxylase Pad1, which catalyzes its conversion to 4-vinylphenol [21, 22], CEN.PK cultures spiked with coumarate did not show reduction in the initial coumarate concentration over a period of 48 h (data not shown). Overexpression of *ARO1*, *ARO7*^*FBR*^, *TYR1* or *TYRC* in addition to *ARO4*^*FBR*^ were all effective at increasing coumarate yields, up to ten-fold relative to TY757 and four-fold relative to TY920 (Fig. 2 and Additional file 1: Figure S1). However, with final (96 h) titers averaging 120 μM for all four strains, no statistically significant difference was observed between these four strategies on coumarate production.

In strain TY985 overexpressing *ARO1,* a decrease in DHS concentration and increases in shikimate and chorismate concentrations relative to TY920 are consistent with the expected increase in Aro1 activity. However, strain TY985 did not produce more total specific carbon downstream of DHS inclusively, and after the end of log phase (24 h) total specific carbon was down by about 29 % when compared to TY920 (Fig. 3). Most of this reduced carbon was due to low levels of tyrosol and tyrosine (Additional file 1: Figure S1-B), which points to possible down-regulation of the expression of Ehrlich pathway machinery in TY985 [14]. Also, initial increases in the concentrations of shikimate, chorismate, prephenate and also 4HPP were observed at 12 h in strain TY985 when compared with TY920. This initial spike in concentration was then moderated, possibly pointing to a robust regulation of intracellular metabolite concentrations.

Overexpression of *ARO7*^*FBR*^ (TY952) was effective at increasing the prephenate pool and depleting the chorismate pool as expected (Fig. 2 and Additional file 1: Figure S1-C). No significant change was observed in the profiles of 4HPP, tyrosine or tyrosol in TY952 when compared with TY920 (Additional file 1: Figure S1-C).

*TYR1* and *TYRC* overexpression had generally the same effect even though they were cloned under different constitutive promoters (*TDH3*prom-*TYRC*, *TEF1*prom-*TYR1*; Additional file 8: Table S1). Both contributed to overall higher levels of 4HPP (Fig. 2 and Additional file 1: Figure S1-D) when compared to strain TY920. The increases in 4HPP were not reflected in tyrosine, but a three-fold improvement in coumarate production was seen by 48 h. The extra pull on prephenate was reflected in a net decrease in phenylalanine measured, but tryptophan concentrations did not change.

### Engineering the host core metabolism for improved precursor and cofactor pools

#### Model-guided platform strain design

In addition to localized changes affecting enzyme activity and regulation of the aromatic amino acid biosynthesis pathway, global changes to yeast core metabolism could be beneficial by improving availability of the precursors PEP and E4P, reducing carbon waste to competing by-products, and shifting cofactor pools to favour product biosynthesis. To this end, the iMM904 model of yeast metabolism [16] was used along with strain design algorithms targeting tyrosine overproduction from glucose with the reasoning that manipulations to core metabolism that result in improved tyrosine production could be equally valuable for many products deriving from tyrosine or other aromatic amino acids. For central metabolites like tyrosine, network complexity and redundancy frequently require several concurrent knockouts in order to obtain a growth-coupled design. Because it uses a global search, OptKnock [17] is computationally intensive and therefore limited to searching a relatively small number of simultaneous knockouts in genome-scale models. OptKnock was not able to find a growth-coupled solution for tyrosine after searching up to four knockouts, therefore the Genetic Design by Local Search (GDLS) [18] algorithm was used to expand the search for higher numbers of knockouts. GDLS by definition does not necessarily find a global optimum, so its solutions are dependent on the quality of initial conditions provided to the algorithm. When no initial conditions are provided, GDLS begins its search from the wild-type model, i.e. with all reactions in place. Under these conditions, GDLS was unable to find a tyrosine producing design when searching up to ten reaction knockouts. To address this challenge, GDLS was first run with chorismate production as the target, and the resulting solution was used as an initial condition for a second iteration of GDLS with tyrosine as the target. This two-step approach resulted in a growth-coupled strain design that produces tyrosine at up to 60 % of the theoretical yield (Additional file 2: Figure S2).

**Fig. 4:**
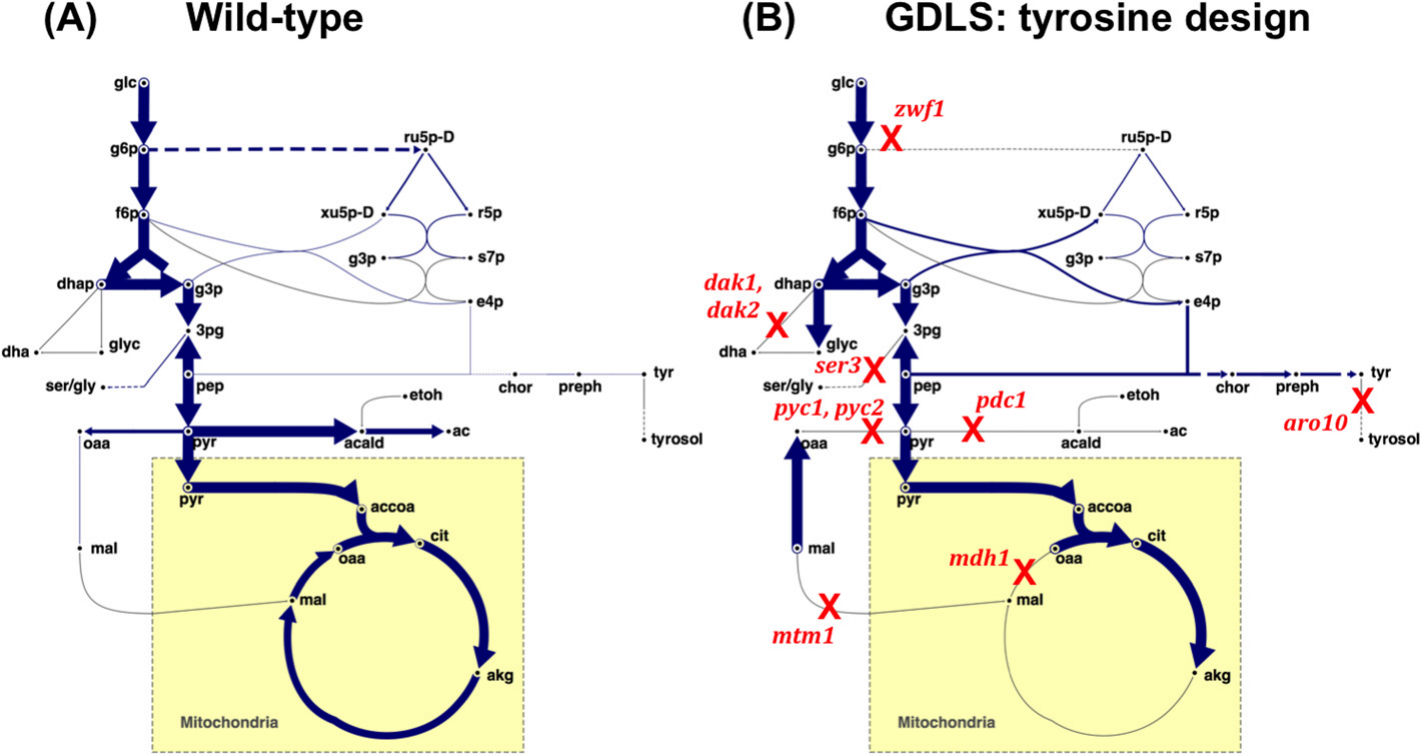
An overview of an *in silico* strain design for growth-coupled tyrosine production. Metabolic fluxes for wild-type **a** and mutant **b** strains were predicted by maximizing biomass production using the iMM904 model during respiratory growth on glucose. Knockouts obtained using the GDLS strain design algorithm are shown in *red* font. The flux distributions are visualized using Omix Visualization software [54], and *arrow* width correlates to predicted flux. Reaction edges carrying no flux are shaded *grey*

The knockout strategy proposed by GDLS combines multiple simultaneous strategies for directing flux towards tyrosine (Fig. 4). GDLS, like many strain design algorithms, is based on the search for strategies resulting in a stoichiometrically growth-coupled strain, meaning that export or accumulation of the desired target is necessary in order to reach the optimal growth state. An important implication of this is that all proposed manipulations are intended to be implemented as a complete set and the resulting strain must be adapted in exponential growth phase to reach a near-optimal growth phenotype. Practically, however, implementation of all proposed manipulations may not be possible in many situations. In our case, for example, knockout of the pyruvate carboxylase reaction (gene: *PYC1*, *PYC2*, reaction ID: PC) was shown to completely prevent production of oxaloacetate during growth on glucose, necessitating supplementation of aspartate to the medium [23]. As a result of this limitation, we evaluated the individual predictions provided by GDLS for their physiological contribution to improved tyrosine production. Knockout of the aromatic amino acid degradation pathway is achieved by removing the phenylpyruvate decarboxylase (*ARO10,* reaction ID: PPYRDC), a strategy that has already been explored experimentally [15]. Because this strategy was already well established, deletion of *ARO10* was implemented in all of our design strains. In our hands, in a strain overexpressing a tyrosine insensitive DAHP synthase, deletion of *ARO10* resulted in at least 4 times less tyrosol and 1.5 times more coumarate than the wild-type background after 48 h (data not shown). Further, knockouts to the pyruvate decarboxylase reaction (*PDC1*, PYRDC), pyruvate carboxylase, mitochondrial malate dehydrogenase (*MDH1*, MDHm), and the malate mitochondrial transporter (*MTM1*, MALtm) were proposed by the GDLS solution. All of these mutations would minimize carbon flux below the pyruvate node, possibly promoting aromatic amino acid biosynthesis derived from the precursors PEP and E4P, but experimental implementation of these knockouts would have introduced many known auxotrophies not predicted by the model stoichiometry alone [23, 24]. As a strategic substitute for these deletions, we selected a point mutation of the main pyruvate kinase isoform *CDC19* previously shown to reduce that enzyme’s activity and result in accumulation of PEP [25]. In addition, the GDLS design eliminates a competing drain on glycolytic flux with the knockout of the 3-phosphoglycerate dehydrogenase (*SER3*, PGCD), the first step in serine and glycine biosynthesis. This knockout is predicted to not result in auxotrophy because of the existence of an alternative synthesis route from alanine via glyoxylate aminotransferase [26]. Finally, the knockout of glucose 5-phosphate dehydrogenase (*ZWF1*, G6PDH2) has the potential to improve both precursor availability and cofactor pools. In particular, the *ZWF1* knockout was found to be important to achieve complete growth-coupling of tyrosine production (Additional file 2: Figure S2). If the design is implemented with *ZWF1* still intact, tyrosine export is predicted to vary over a range due to alternate optimal solutions. This is due to the fact that either tyrosine or phenylalanine can be exported equally well in this scenario, according to the steady-state model (not shown). In light of this, the potential impacts of *ZWF1* knockout on tyrosine production are two-fold. First, in the absence of the oxidative pentose phosphate pathway, the important biomass precursors ribose 5-phosphate (R5P) and E4P must be obtained through a reversal of the non-oxidative pentose phosphate pathway. Under low or absent seduheptulose 1,7-bisphosphatase (*SHB17*) activity as predicted by the iMM904 model, E4P must be produced in excess in order to meet biomass requirements for R5P. This strategy has the potential to improve E4P availability for aromatic amino acid production. Second, the *ZWF1* knockout affects the cell’s ability to regenerate cytosolic NADPH pools, which in steady-state would promote prephenate dehydrogenase flux, catalyzed by the NADPH-generating Tyr1. Finally, the model suggests a knockout of dihydroxyacetone kinase (*DAK1* and *DAK2,* DHAK), which prevents a futile cycle allowing the NADP^+^-dependent glycerol dehydrogenase from regenerating NADPH.

### Effect of *ZWF1* knockout on flux to aromatic amino acid and coumarate

In order to test the changes to core metabolism for their impact on aromatic amino acid and coumarate production, we tested a knockout of *ZWF1*. Because manipulation of NADPH pools has the potential for far-reaching effects throughout metabolism, it was desirable to test Zwf1^−^ strains along with mechanisms to alleviate the effects of NADPH depletion. Therefore, overexpression of NADP^+^-dependent Tyr1 (strain TY1040) was predicted to alleviate NADPH depletion and promote tyrosine production while NAD^+^-dependent TyrC (TY1041) would not. Additionally, methionine supplementation has been shown to be necessary for growth of Zwf1^−^ S288C strains [27], presumably because it reduces the cellular demand for NADPH during amino acid synthesis. As all Zwf1^−^ strains tested in this study were able to grow in minimal medium, the methionine auxotrophy that is associated with the *ZWF1* knockout was not observed. However, knocking out *ZWF1* did result in a reduction of the specific maximum growth rate, growth yield and glucose uptake rate by about half for both strains with respect to their Zwf1^+^ counterparts. Methionine supplementation was able to partially recover maximum specific growth rate of both Zwf1^−^ strains (Table 2).

**Fig. 5:**
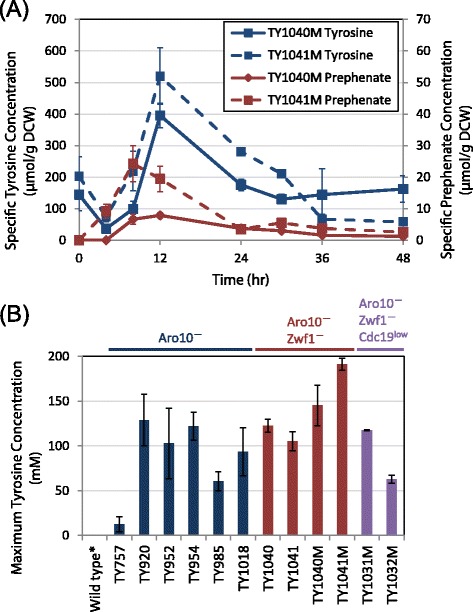
Intracellular tyrosine regulatory effects and accumulation. **a** Intracellular tyrosine and prephenate concentration for strains TY1040 (*zwf1Δ [ARO4* ^*FBR*^ *TYR1]*) and TY1041 (*zwf1Δ [ARO4* ^*FBR*^ *TYRC]*) are shown during growth on methionine-supplemented YNB. **b** The maximum intracellular tyrosine concentration observed for all strains. The wild-type tyrosine concentration was reported previously for *S. cerevisiae* S288C*. Error bars* in both panels signify 95 % confidence intervals based on three biological replicates

In Zwf1^+^ strains overexpressing either *TYR1* or *TYRC,* 4HPP plateaued at the end of log phase (24–30 h) before being depleted in stationary phase (Fig. 2, Additional file 3: Figure S3-A and Additional file 4: Figure S4-A). When *ZWF1* was knocked out and *TYR1* overexpressed, 4HPP dropped by about five-fold, as did prephenate/phenylpyruvate and coumarate (Additional file 3: Figure S3-A). With *TYRC* overexpression, on the other hand, 4HPP was overall lower but continued to accumulate past 36 h and by 48 h was higher than for its Zwf1^+^ counterpart (Additional file 4: Figure S4-A). Starting levels of tyrosine were higher for both strains and when cultures were supplemented with methionine, they showed the highest intracellular tyrosine levels of any tested, reaching 395 and 520 μmol/g DCW, respectively, although these levels were not sustained (Fig. 2, Additional file 3: Figure S3-B and Additional file 4: Figure S4-B). The spike in tyrosine concentration for strains TY1040 and TY1041 at 12 h coincided with a decrease in prephenate concentration beginning at the same time point (Fig. 5a), despite chorismate levels remaining relatively constant. This result is consistent with allosteric inhibition of Aro7 by tyrosine. DHS was not significantly higher for Zwf1^−^ than for Zwf1^+^ strains when overexpressing *TYRC*, but was ten-fold greater when overexpressing *TYR1* (Fig. 2). The maximum intracellular tyrosine concentration observed at any time point for all strains can be seen in Fig. 5b.

Supplementation of methionine led to improvements in total carbon downstream of 4HPP inclusively for all Zwf1^−^ strains: an 84 % boost for *TYR1* (197 to 363 μmol/g DCW) and a 20 % increase for *TYRC* (from 489 to 600 μmol/g DCW) (Fig. 3). Coumarate production was lower for Zwf1^−^ strains compared to Zwf^+^ strains; between the two Zwf^−^ strains, both with and without methionine supplementation, the TyrC overexpressing strain (TY1041) had a better coumarate output than the Tyr1 strain (TY1040) (Fig. 2, Additional file 3: Figure S3-B and Additional file 4: Figure S4-B). This was contrary to the hypothesis that in a Zwf1^−^ background overexpression of NADP^+^-dependent Tyr1 would help to alleviate NADPH depletion and promote tyrosine production while overexpression of NAD^+^-dependent TyrC would not.

### Effect of cdc19^T21E^ on flux to aromatic amino acid and coumarate

The strain designs obtained by GDLS for tyrosine overproduction are intended to capture metabolic effects from a very broad perspective, but there are many limitations to the practical implementation of strain designs obtained using these models. In particular, because regulatory and metabolite concentration information is not well captured in such models, many of the design components are either unnecessary or biologically infeasible. For example, knockout of the PYRDC reaction (through deletion of *PDC1*, *PDC5*, and *PDC6*) is allowable in the iMM904 model at steady-state, while yeast strains with these mutations are not able to grow on glucose [28, 29]. This error is in part because the Crabtree effect and its regulatory implications are not captured in this model. DAHP synthesis together with the final reaction carried out by Aro1 account for <1 % of PEP flux consumption in growing yeast cells [30]. The balance is consumed by the major pyruvate kinase isozyme Cdc19, which catalyzes the majority of pyruvate production during growth on glucose. Many of the deletions obtained by GDLS had the effect of reducing carbon flux beyond the pyruvate node. One potential way to mimic this design choice without implementing these knockouts is the knockdown of pyruvate kinase achieved by a T21E mutation to the *CDC19* gene [25]. This mutation mimics the phosphorylated form of the enzyme and was shown to have impaired activity and a corresponding increase in intracellular PEP concentration [25].

To test the contribution of increased PEP pools toward aromatic amino acid production, overexpression of *TYR1* or *TYRC* along with *ARO4*^*FBR*^ was moved into a Zwf1^−^ Cdc19^low^ strain, giving rise to strains TY1032 and TY1031, respectively. Neither TY1031 nor TY1032 grew in the absence of methionine; therefore comparisons drawn below are with respect to Zwf1^−^ Cdc19^+^ counterpart strains grown in the presence of methionine.

Specific maximum growth rate was not higher for Zwf^−^ Cdc19^low^ strains than it was for Zwf^−^ Cdc19^+^ strains; however, growth yield was improved, approaching levels observed in the Zwf1^+^ Cdc19^+^ background (without methionine; Table 2). We expected to observe some evidence of PEP accumulation and/or a decrease in pyruvate or its by-products. Although no direct differences in PEP or pyruvate were measured, indirect effects observed bear out the presence of the mutation. For both the *TYR1* and *TYRC* cases, the addition of the *cdc19*^*T21E*^ mutation led to a drop of about 50 % in total carbon downstream of 4HPP; from 343 down to 157 μmol/g DCW for TY1032 and from 600 to 287 μmol/g DCW for TY1031 (Fig. 3). For Cdc19^low^ strains (TY1032 and TY1031), 4HPP began to disappear as the strains reached the end of log growth (24–30 h) whereas for TY1041 it was observed to be still accumulating by 48 h (Fig. 2, Additional file 3: Figure S3-C and Additional file 4: Figure S4-C). For both cases with the pyruvate kinase mutant, tyrosine concentrations were overall much lower than Zwf^−^ Cdc^+^ levels and showed a steady decline from the start of growth. Coumarate was lower for both, but lower from the start of growth for *TYRC* whereas for *TYR1* it only dropped off from Zwf1^−^ Cdc19^+^ levels towards the end of log. With respect to overflow metabolism, the *TYRC* strain showed considerable changes (Additional file 5: Figure S5-C and Additional file 6: Figure S6-C). Whereas TY1041 produced and accumulated high amounts of acetate in the culture supernatant, TY1031 produced as much but began to re-assimilate it from the start of log phase and consumed virtually all of it by 48 h. This response profile resembles that generally observed for the Zwf1^+^ Cdc19^+^ strains (Additional file 6: Figure S6-A). TY1031 also produced more ethanol than TY1041. Glycerol profiles for both TY1031 and TY1032 were similar to those observed for their Zwf1^−^ Cdc19^+^ counterparts except that initial concentrations were higher (Additional file 5: Figure S5-C and Additional file 6: Figure S6-C).

## Discussion

We created a series of model-driven modifications to wild-type CEN.PK yeast to divert carbon flux for tyrosine overproduction, monitoring nineteen metabolites over the course of shake-flask fermentations on glucose. Using a targeted metabolomics time-course strategy we sought not only to evaluate our ability to overproduce tyrosine but also to identify pathway bottlenecks that might present new potential engineering targets.

Cytosolic tyrosine concentration in wild-type yeast has been previously reported at 0.5 mM [7]. The maximum intracellular tyrosine measured for our Aro10^−^ base case was 34 μmol/g DCW at 72 h (Fig. 5b). This corresponds to a concentration of 19 mM in the cell, a 38-fold increase over the reported value. Although these are different strains, it is likely that this tyrosine accumulation is predominantly due to partial disruption of the tyrosine degradation pathway by knocking out *ARO10*. With *ARO4*^*FBR*^ overexpressed in the Aro10^−^ strain, a maximum tyrosine value of 351 μmol/g DCW or 129 mM was obtained in the cytosol after 30 h, a further improvement of nearly 7-fold. Zwf1^−^ TY1041 expressing *TYRC ARO4*^*FBR*^ in the presence of methionine produced our highest recorded tyrosine level of 520 μmol/g DCW or 192 mM in the cell, a further gain of 1.5-fold (a 384-fold total increase over wild-type); however, it is clear that the manipulation of NADPH/NADP^+^ ratios to promote tyrosine formation is not straightforward, as some *ZWF1* deletion strains exhibited little or no improvement over Zwf1^+^ strains. We hypothesize that the effect of this deletion on NADPH pools must be carefully controlled in order to balance improved favourability of prephenate dehydrogenase flux against creation of a separate cofactor limitation at shikimate dehydrogenase, for example by using a controlled knock-down of Zwf1 expression.

Although coumarate levels seem to respond to improvements in tyrosine concentration at low fluxes, the TAL enzyme appeared to be quickly saturated, resulting in high carbon losses in the aromatic amino acid pathway. All four first-generation attempts to improve on the chassis strain case (strains TY985, TY952, TY1018 and TY954) resulted in a virtually identical increase in coumarate (Fig. 2) despite affecting the total carbon in the aromatic pathways in vastly different ways.

In our hands, overexpression of the *ARO4*^*FBR*^ variant alone in an Aro3^+^ Aro4^+^ Aro10^−^ strain increased tyrosine and total carbon measured in the shikimate and aromatic pathways by more than five-fold with respect to our control strain.

Other than prephenate dehydrogenase, only one other reaction in tyrosine biosynthesis involves the reducing equivalent NADPH directly, namely the NADPH-dependent 5-dehydroshikimate (DHS) reductase activity of Aro1. Because of this, tyrosine biosynthesis can be considered NADPH neutral, apart from the production of glutamate as substrate for the final aminotransferase reaction by Aro8. A third reaction, chorismate synthesis by Aro2, involves NADPH only indirectly, requiring it for the reduction of its cofactor FMN but not consuming it [31]. The NADPH deficiency resulting from deletion of *ZWF1* limited carbon flux at the NADPH-dependent DHS reductase activity of Aro1. This resulted in a build-up of DHS and backed carbon up to PEP, which also showed an accumulation. It was expected that partial relief of NADPH deficiency – either by the addition of methionine or by increasing flux through the NADP^+^-dependent prephenate dehydrogenase – would improve the thermodynamic favourability of DHS reduction to shikimate. In all Zwf1^−^ strains, methionine supplementation was able to achieve improved flux through the shikimate pathway, increased tyrosine concentrations, and higher final coumarate titers. Surprisingly, however, overexpression of the non-native NAD^+^-dependent TyrC was found to outperform overexpression of the native NADP^+^-dependent Tyr1 for driving flux through 4HPP. While many prephenate dehydrogenases are inhibited by tyrosine, the Tyr1 from *S. cerevisiae* is only known to be regulated at the transcriptional level [13]. TyrC from *Z. mobilis*, on the other hand, is known to be feedback-insensitive to tyrosine [12]. This result could reflect a previously undescribed inhibition of the Tyr1 from *S. cerevisiae* by tyrosine.

An observed decrease in E4P in Zwf1^−^ strains may have been the result of a bottleneck in the reverse of the non-oxidative pentose phosphate pathway when *ZWF1* is deleted. To achieve predicted improvements to flux through E4P, it may be necessary to overexpress *TKL1* as demonstrated by Curran et al. for the production of muconic acid [32]. The initial high levels of tyrosine suggest that the cell did try to overcome the deficiency with Tyr1 activity. However, this resulted in shutdown of Aro7 as evidenced by the early spike in chorismate. The growth rate, growth yield and glucose uptake rate were reduced by about half (Table 2) in the *ZWF1* knockout strains, and we measured overall lower levels of all three overflow metabolism products (Table 3) monitored for the *TYR1* case. This observation combined with high PEP measured in these strains may have signalled a slowdown in glycolysis. Furthermore the reduction in re-assimilation rates of ethanol, acetate and glycerol suggested that the diauxic shift normally signalled by glycolytic intermediates did not occur.

The incorporation of the *cdc19*^*T21E*^ mutation did not improve overall flux through the aromatic amino acid biosynthesis pathway, resulting in a further drop in carbon downstream of 4HPP inclusively for both *TYR1* and *TYRC* strains, by about half compared to their Zwf1^−^ Cdc19^+^ counterparts. The *cdc19*^*T21E*^ mutation reigned in the conversion of PEP to pyruvate, and this restricted the amount of substrate carbon in the form of acetaldehyde the *TYRC* strain could send to Ald6 to generate NADPH. As a result, the *TYRC* case showed a greater than twenty-fold increase in DHS due to the NADPH requirement of the DHS reductase activity of Aro1.

The use of constraint-based modelling as a platform for optimization-based strain design has generated considerable interest for the past several years, but only a few examples of practical success using these approaches have been demonstrated, particularly for secondary metabolite production. One possible explanation for this is the relatively high energetic and material costs of secondary metabolites, which frequently require several reaction knockouts in combination in order to achieve a growth-coupled phenotype. Because most constraint-based models do not fully incorporate regulatory and thermodynamic limitations, model prediction inaccuracies are inevitable, and a higher number of combined knockouts is increasingly likely to become experimentally infeasible. In this study, it was determined based on previous experimental results for individual knockouts [23, 24] that concurrent implementation of all GDLS design strategies for tyrosine production would not be feasible. Specifically, this model inaccuracy could be attributed to the inability to capture major regulatory shifts in *S. cerevisiae* metabolism from fermentative to respiratory growth (i.e. the Crabtree effect) using current models. In this study, we demonstrate that computational strain design algorithms depending on growth-coupling, such as GDLS, can still provide valuable insight into broad, non intuitive design strategies, but interpretation and implementation of these strategies is not straightforward. The deletion of *ZWF1* for improved aromatic amino acid pathway flux has been used previously in combination with overexpression of transketolase *TKL1* [32] to improve availability of the precursor E4P. The constraint-based modelling approach used here confirmed the utility of that manipulation for improving E4P pools and also identified it as a tool for shifting cytosolic NADPH pools in favour of tyrosine production, reducing carbon loss to phenylalanine.

## Conclusions

In this study, we systematically evaluated both rational pathway engineering and model-driven strain design strategies for the improvement of tyrosine production. In Aro10^−^ strains overexpressing deregulated aromatic amino acid biosynthesis enzymes, this approach demonstrated possible cofactor limitation at prephenate dehydrogenase, indicated by accumulation of prephenate by these strains. Genome-scale modelling identified a *ZWF1* knockout strategy as a potential solution to this by changing NADPH/NADP^+^ ratios in the cytosol to make the prephenate dehydrogenase reaction more thermodynamically favourable. Our results indicate that this strategy is able to improve tyrosine accumulation *in vivo*, but careful control of its effects on cofactor pools is critical to avoid unwanted effects. Additionally, our findings confirm the importance of transcript- and protein-level deregulation of the aromatic amino acid pathway and the complete removal of potential degradation pathways for sustained diversion of carbon flux through tyrosine toward higher value secondary products.

## Materials and methods

### Strains and plasmids

Full descriptions of the *Saccharomyces cerevisiae* strains and plasmids used in this study are given in Additional file 8: Table S1. *Escherichia coli* DH5α was used to maintain and propagate plasmids. *E. coli* was grown at 37 °C and 200 rpm in LB medium supplemented with 100 μg/mL of ampicillin. *S. cerevisiae* was grown at 30 °C and 150 rpm in either the rich medium YPD or the defined SD medium [YNB supplemented with 2 % (w/v) glucose] [33]. When required, 200 μg/mL geneticin (G418) and 200 μg/mL hygromycin were added to YPD, and SD medium was supplemented with amino acids to complement specific auxotrophic requirements [34].

### Plasmid construction

The DNA assembler method [35] was used to construct the plasmids used in this study. The different DNA parts were amplified by PCR, run on agarose gel and individually purified using Qiagen Gel Purification kit (Valencia, CA, USA). All primers used in this study are listed in Additional file 9: Table S2. DNA parts (promoter, gene, terminator) were pooled with a linearized plasmid and transformed in the appropriate yeast singly auxotrophic strain by electroporation, as described by Shao et al. [35]. Assembly was selected for by growth on minimal medium, and the resulting plasmids were recovered from yeast and transformed into *E. coli* for maintenance. Sanger sequencing confirmed correct assembly of the parts. Promoters and terminators required for assembly were amplified from *S. cerevisiae* CEN.PK genomic DNA. The genes coding for the aromatic amino acid synthesis enzymes were assembled in centromeric shuttle plasmids derived from pGREG506 and pGREG503 [36]. The feedback inhibition-resistant version of the DAHP synthase *ARO4* was obtained by introducing the K229L mutation by PCR, using *S. cerevisiae* CEN.PK genomic DNA as template [11, 37]. Similarly, the G141S mutation was introduced into *ARO7* to create a feedback inhibition-insensitive version of the chorismate mutase [11]. Native coding genes for *TYR1* and *ARO1* were PCR-amplified from *S. cerevisiae* CEN.PK genomic DNA. *TYRC* from *Z. mobilis* and *TAL* from *R. sphaeroides* were synthesized and codon-optimized for *S. cerevisiae* by DNA 2.0 (Menlo Park, CA, USA). All heterologous genes were cloned under different constitutive yeast promoters: *ARO4*^FBR^ under *pFBA1* in plasmid pTY978, *ARO1* under *pPYK1* in pTY502, *ARO7*^FBR^ under *pPDC1* in pTY688, *TYR1* under *pTEF1* in pTY1035, and *TYRC* under *pTDH3* in pTY500. *TAL* was assembled under *pPMA1* into a *2 μ* shuttle vector derived from pYES2 (Life Technologies, Carlsbad, CA, USA), giving rise to pTY350. Plasmids pTY338 and pTY51 served as empty vector controls.

### Gene knockouts and knock-ins

Chromosomal gene knock-outs were done by homologous recombination, using antibiotic marker-containing disruption cassettes created by PCR, as described by Gueldener et al. [38]. Integration cassettes contained two 40-nt regions of homology corresponding to the 5′ and 3′ ends of the target locus. The *loxP*-flanked *kanMX* and *loxLE*/*RE*-flanked *hphNT1* cassettes were amplified from the pUG6 and pZC3 vectors, respectively [38, 39]. The mutant allele coding for Cdc19^T21E^ was amplified from genomic DNA extracted from W303-based strain JR201, provided courtesy of J. Rabinowitz [25], using primers upstream of the gene and downstream of a *kanMX* marker. The resulting PCR cassette was integrated into CEN.PK111-61A by homologous recombination. Transformations into yeast of knock-out or knock-in cassettes (as well as all plasmids) were performed by the lithium acetate method [40]. After transformation, cells were plated on YPD agar containing 200 μg/mL G418 and/or hygromycin, as appropriate. Presence of an antibiotic marker linked to a gene knockout or insertion was confirmed by PCR. Sanger sequencing validated the presence of the *CDC19* mutant allele (61 A > G + 62 C > A + 63 C > G). Single mutations were made in one of the compatible mating types of the triple auxotroph (*ura3 leu2 his3*) haploid wild-type, CEN.PK111-61A *MATα* or CEN.PK111-5B *MATa*. Mutations were compiled by mating single mutation strains or by transforming a second deletion cassette into an existing knockout strain. Strains H703 and H712 were mated to generate H919. *ARO10* was deleted with a *loxLE*-*hphNT1*-*loxRE* cassette in strain H749 to generate H1045. Strains H749 and H919 were mated to generate H876. The antibiotic marker was removed from H703 only, using the Cre recombinase plasmid pSH47 [38], to generate strain H837. Different three-plasmid combinations were transformed into host strains, giving rise to the “TY” test strains listed in Table 1.

### Model-guided strain design

Genome-scale constraint-based metabolic modelling [41] was used to predict and evaluate the impacts of metabolic gene deletion on the metabolic phenotype. Simulations were done on the *in silico* reconstruction of yeast metabolism iMM904 [16] using the COBRA Toolbox v2.0 [42] in Matlab using CPLEX ILOG for optimization. All uptake fluxes during strain design simulations were set at zero except for glucose and oxygen exchange, which were set with lower bounds of −10 mmol/gDCW/h. The OptKnock strain design algorithm [17] was implemented in MATLAB, searching up to four simultaneous reaction deletions with the outer objective of maximizing an artificial cytosolic L-tyrosine exchange flux. Genetic Design by Local Search (GDLS) [18] was performed using an in-house implementation with the same boundary conditions and either cytosolic L-tyrosine or cytosolic chorismate exchange fluxes as the outer objective. GDLS was run with a neighbourhood size of 2 and a maximum of 10 knockouts. Simulations were tuned to mimic respiratory growth on glucose, with glucose and oxygen uptake set at a ratio of 1:1. These conditions are generally able to reproduce biomass and by-product yields observed during steady-state respiratory growth [43].

In order to simulate wild-type flux through the oxidative pentose phosphate pathway for Fig. 4, additional changes were required. First, the cytosolic isocitrate dehydrogenase, *IDP2* (reaction ID: ICDHy), is knocked out to reflect its downregulation in glucose conditions [44]. Second, the oxoadipate/α-ketoglutarate mitochondrial antiporter was added to allow transport of cytosolic α-ketoglutarate into the mitochondria [45]. Finally, the cytosolic acetaldehyde dehydrogenase *ALD6* (reaction ID: ALDD2y) was proportionally limited to 16 % of the glucose uptake rate to reflect its physiological contribution to cytosolic NADPH supply [43].

### Analysis of metabolites

#### Metabolites extraction

For each strain tested, three pre-cultures were seeded from individual colonies streaked out on minimal medium. Pre-cultures were grown overnight and then used to inoculate 40 mL of YNB with 2 % (w/v) glucose in a 250-mL shake-flask to a starting OD_600_ of 0.05. Growing cultures were then sampled at regular intervals and optical densities were read using a TECAN Infinite 200 PRO in a 96-well plate format, diluting the culture ten-fold into 200 μL of fresh medium.

One mL of culture broth was centrifuged at 21,000 × *g* for 3 min and the supernatant was frozen at −20 °C. An aliquot of 400 μL of the culture supernatant was used for glucose, organic acid and ethanol analysis, and another 400 μL of supernatant was extracted with 2 volumes of ethyl acetate (EtAc) for analysis of non-polar aromatic compounds. Prior to analysis, the EtAc extracts were dried down to completeness in a SpeedVac with no heating. The dried extracts were suspended in 40 μL of 50 % (v/v) acetonitrile (ACN) and 0.05 % (v/v) trifluoroacetic acid (TFA). Intracellular metabolites were obtained as follows based on extraction studies by Villas-Boas et al. and Crutchfield et al. [46, 47]. One mL of culture broth was removed and immediately quenched by adding it to 5 mL of pure methanol (MeOH) chilled in a bath of ethanol and dry ice. The mixture was immediately centrifuged at −9 °C and 3000 × *g* for 5 min. The supernatant was discarded and the cell pellets were suspended in 400 μL 80 % (v/v) MeOH pre-chilled at −20 °C, and then incubated on ice for 15 min. The mixtures were then centrifuged at 16,000 × *g* for 5 min at 4 °C. Supernatants were removed and set aside, and the pellets were extracted again with a second volume of 400 μL 80 % MeOH. The pooled extracts were dried to completeness in a SpeedVac with no heating. The dried extracts were suspended in 200 μL of 0.1 % (v/v) formic acid (FA) for HPLC analysis.

#### HPLC analysis

A 10 μL aliquot of the non-extracted supernatants was injected, using a Finnigan Surveyor HPLC system, onto an Aminex HPX-87H column (7.8 × 300 mm, 9 Å, Biorad) heated to 65 °C. Glucose, glycerol, acetate and ethanol were resolved isocratically in 5 mM H_2_SO_4_ at 0.6 mL/min. Metabolites were identified and quantitated by a refractive index detector set to 35 °C using standards.

The EtAc-extracted supernatants were analyzed on an Eclipse XDB-C18 column (4.6 × 150 mm, 5 μm, Agilent), using an Agilent 1200 HPLC system equipped with a photodiode array detector. Metabolites from 5 μL of the concentrated extracts were separated at 1 mL/min using a gradient method where mobile phase A was 0.1 % (v/v) TFA in water and B was 0.1 % (v/v) TFA in MeOH. The gradient was as follows: 0–0.5 min 20 % B, 0.5–10 min 20–50 % B, 10.5–18.5 min 50–98 % B, 18.5–21.5 min 98 % B. Various UV wavelengths were used to follow, in order of elution, tyrosol (276 nm), chorismate (276 nm), 4-hydroxyphenylacetate (276 nm), 4-hydroxyphenylacetaldehyde (285 nm), 4-hydroxyphenylpyruvate (304 nm), coumarate (310 nm), tryptophol (276 nm), and prephenate/phenylpyruvate which are indistinguishable (290 nm). Prephenate and phenylpyruvate were measured as a mixed peak by RP-HPLC/PDA; however, the kinetics of the signal (its maximum typically coinciding with that of chorismate and being exhausted completely within 36 h) suggests that the peak consisted predominantly of prephenate. Thus, we herein refer to prephenate/phenylpyruvate numbers as though they connote the metabolite prephenate.

Cell extracts were analyzed by single-reaction monitoring (SRM) mass spectrometry using a Thermo LTQ-MS equipped with an electrospray ionization source and a Surveyor HPLC system. Positive mode was used to detect the aromatic amino acids, which were first resolved on a Zorbax Eclipse XDB-C18 column (4.6 × 30 mm, 1.8 μm, Agilent). The following gradient was used for separation of the metabolites: 0–1 min 3 % B, 1–10 min 3–97 % B, 10–12 min 97 % B, where mobile phase A was 0.1 % (v/v) FA in water and B was 0.1 % (v/v) FA in MeOH. The flow rate was set at 100 μL/min with the spray voltage set to +4 kV and the sheath gas at 5. Tyrosine was detected as the transition from +182 to +165 m/z, using a collision induced dissociation (CID) energy of 15 and isolation width of 1.5 m/z. L-Phenylalanine was detected as the transition from its parent ion +165 to +120 m/z, using a CID of 20. L-Tryptophan was monitored as the transition from +205 to +188 m/z, using a CID of 15. Standard curves were run for quantitation.

Negative mode was used for detection of phosphorylated sugars and shikimate pathway intermediates. A Fast Acid Analysis column (7.8 × 100 mm, 9 Å, Biorad) heated to 65 °C was used with 0.1 % (v/v) FA in water, running isocratically at 0.6 mL/min. Ten μL of extract was injected and the flow was split post-column to about 100 μL/min to the ESI source. The spray voltage was set to −3.6 kV and the sheath gas was set to 5. Although PEP and E4P co-elute they could be analysed by monitoring the transition of −167 to −79 m/z for PEP and the transition of −199 to −97 m/z for E4P. Isolation width of 1.5 m/z and CID of 35 was used for all metabolites. Shikimate and DHS co-eluted as well. The transition of −173 to −155 m/z was used for shikimate, while DHS was monitored as the transition of −171 to −129 m/z. Pyruvate could not be measured by SRM because the LTQ is limited in its ability to trap and isolate ions smaller than about 150 m/z. Therefore, pyruvate was measured in full scan mode in the low mass range using the transition from its parent ion −87 m/z to itself with no collision energy. Standard curves were run for identification and quantitation.

#### Calculations

Dry cell weight (DCW) was determined by multiplying OD_600_ values by a conversion factor of 2.01 mg DCW/mL/OD_600_, a relationship determined in-house from *S. cerevisiae* CEN.PK grown in minimal medium.

Maximal growth rate μ_MAX_ (1/h) was calculated using least-squares fitting during the exponential growth phase using the Doubling Time website [48]. Growth yield Y_X/S_ was calculated as the maximum grams of DCW per grams glucose consumed. Average final titer was estimated based on a parameter-3 sigmoidal fit to data points using SigmaPlot11.0. Rate determinations were made for individual clones and the average value with 95 % confidence interval is reported.

Specific carbon totals were estimated as follows. Specific carbon was added up at each time point up to 48 h for metabolites downstream of a given enzyme modification. The data were plotted against time in SigmaPlot 11.0 and fitted with a 2-parameter exponential rise to maximum curve fit. Total specific carbon reported was taken from the fit at 48 h and the error reported is the error of the fit.

ANOVA were run on all data sets compared. A Student’s *t*-test was performed when values were compared at only a single time point. The null hypothesis was rejected when *p* < 0.05.

The following values were used in the estimation of cytosolic concentrations: 160 μm^3^ (or 1.6 × 10^−13^ L) for the volume of the cell [49] and 60 pg for the mass of a cell [50]. Thus, we assume 1.7 × 10^10^ cells/g DCW and an intracellular volume of 2.7 mL per g DCW.

Estimation of the reaction change in Gibbs free energy (*ΔG*_*γ*_) for each step in the tyrosine biosynthesis pathway (see Additional file 7: Figure S7) was done as follows. Standard *ΔG*_*γ*_ values for each step were calculated using the component contribution method [51] at an assumed cytosolic pH of 6.5 and at a temperature of 25 °C. *ΔG*_*γ*_ for each reaction was calculated using the standard relationship

$$ \varDelta {G}_{\gamma }=\varDelta {G}_{\gamma }{}^0 + R\times T \times \ln Q $$

where R is the gas constant 8.314 J/mol/K, T is the temperature in degrees Kelvin, and Q is the reaction quotient, or the product of the concentrations of all participating metabolites raised to their stoichiometric coefficients. For a two reactant and two product reaction in the form

$$ aA+bB\leftrightarrow cC+dD $$

the reaction quotient would be calculated

$$ Q=\frac{{\left[C\right]}^c{\left[D\right]}^d}{{\left[A\right]}^a{\left[B\right]}^b} $$

Intracellular concentrations for PEP, E4P, DHS, SHIK, and TYR were calculated as described above and assumed to be equivalent to concentrations in the cytosol. Extracellular concentrations of CHOR, PPH, and 4HPP were calculated as described above, and the cytosolic concentrations of these metabolites were assumed to be equivalent to the extracellular concentrations. Cytosolic concentrations of the cofactors NADPH, NADP, NADH, and NAD were set at 151.1, 20.37, 174.8, and 862.9 mM, respectively, based on literature values for wild-type *S. cerevisiae* during batch growth on glucose-rich minimal media [52]. Cytosolic concentrations of ATP, ADP, and Pi were set at 4.25, 0.93, and 6.6 M, respectively, based on literature values for wild-type *S. cerevisiae* during batch growth on glucose-rich minimal media [53]. All other metabolites were assumed to have a concentration of 1 mM. All extracellular and intracellular metabolite and biomass concentration data are provided in triplicate for each strain over the 96 h fermentation time course as Additional file 10.

## Additional files

Additional file 1: Figure S1. Aromatic amino acid pathway metabolite profiles from deregulation or overexpression of genes impinging on tyrosine biosynthesis plus *TAL* in Aro10^−^ CEN.PK. **a** Overexpression of *ARO4* ^*FBR*^, strain TY920 versus strain TY757. **b** Overexpression of *ARO1* with *ARO4* ^*FBR*^, strain TY985 versus strain TY920. **c** Overexpression of *ARO7* ^*FBR*^ with *ARO4* ^*FBR*^, strain TY952 versus strain TY920. **d** Overexpression of *TYR1* and *TYRC* with *ARO4* ^*FBR*^, strains TY1018 and TY954 versus strain TY920. Dehydroshikimate, shikimate, L-tryptophan, L-phenylalanine, and L-tyrosine were measured intracellularly. Chorismate, tryptophol, prephenate/phenylpyruvate, 4-hydroxyphenylpyruvate, 4-hydrophenylacetaldehyde, 4-hydroxyphenylacetate, tyrosol, and 4-coumarate were measured extracellularly. *Dotted line* indicates allosteric feedback inhibition. Values represent an average of three biological replicates and *error bars* represent 95 % confidence intervals. Additional file 2: Figure S2. Tyrosine-overproducing strain design obtained using GDLS. Product-growth envelopes are shown using the iMM904 model with glucose and oxygen uptake set at −10 mmol/g DCW/h. Whereas the wild-type strain (*black line*) yields no surplus tyrosine at optimal growth, the complete GDLS mutant strain (*red line*) indicates strong growth coupling and a glucose yield near 60 % theoretical. If the design is implemented with *ZWF1* still intact (*blue line*), tyrosine export is predicted to vary over a range due to alternate optimal solutions in which either tyrosine or phenylalanine can be exported equally. Additional file 3: Figure S3. Aromatic amino acid pathway metabolite profiles from overexpression of *TYR1* with *ARO4* ^*FBR*^ in different genetic backgrounds. **a** In Aro10^−^ Zwf1^−^ Cdc19^+^ versus Aro10^−^ Zwf1^+^ Cdc19^+^, strain TY1040 versus TY1018. **b** In Aro10^−^ Zwf1^−^ Cdc19^+^ with and without methionine added to the growth medium, strain TY1040. **c** In Aro10^−^ Zwf1^−^ Cdc19^+^ versus Aro10^−^ Zwf1^−^ Cdc19^low^ with methionine added to the growth medium, strain TY1032 versus TY1040. Dehydroshikimate, shikimate, and L-tyrosine were measured intracellularly. Chorismate, prephenate/phenylpyruvate, 4-hydroxyphenylpyruvate, and 4-coumarate were measured extracellularly. Values represent an average of three biological replicates and *error bars* represent 95 % confidence intervals. *Dashed line* indicates allosteric feedback inhibition. Additional file 4: Figure S4. Aromatic amino acid pathway metabolite profiles from overexpression of *TYRC* with *ARO4* ^*FBR*^ plus *TAL* in different genetic backgrounds. **a** In Aro10^−^ Zwf1^−^ Cdc19^+^ versus Aro10^−^ Zwf1^+^ Cdc19^+^, strain TY1041 versus TY954. **b** In Aro10^−^ Zwf1^−^ Cdc19^+^ with and without methionine added to the growth medium, strain TY1041. **c** In Aro10^−^ Zwf1^−^ Cdc19^+^ versus Aro10^−^ Zwf1^−^ Cdc19^low^ with methionine added to the growth medium, strain TY1031 versus TY1041. Dehydroshikimate, shikimate, and L-tyrosine were measured intracellularly. Chorismate, prephenate/phenylpyruvate, 4-hydroxyphenylpyruvate, and 4-coumarate were measured extracellularly. Values represent an average of three biological replicates and *error bars* represent 95 % confidence intervals. *Dashed line* indicates allosteric feedback inhibition. Additional file 5: Figure S5. Shikimate pathway precursors and overflow metabolites from overexpression of *TYR1* with *ARO4* ^*FBR*^ plus *TAL* in different genetic backgrounds. **a** In Aro10^−^ Zwf1^−^ Cdc19^+^ versus Aro10^−^ Zwf1^+^ Cdc19^+^, strain TY1040 versus TY1018. **b** In Aro10^−^ Zwf1^−^ Cdc19^+^ with and without methionine added to the growth medium, strain TY1040. **c** In Aro10^−^ Zwf1^−^ Cdc19^+^ versus Aro10^−^ Zwf1^−^ Cdc19^low^ with methionine added to the growth medium, strain TY1032 versus TY1040. Erythrose 4-phosphate, phosphoenolpyruvate and pyruvate were measured intracellularly. Glycerol, acetate and ethanol were measured extracellularly. Values represent an average of three biological replicates and *error bars* represent 95 % confidence intervals. Additional file 6: Figure S6. Shikimate pathway precursors and overflow metabolites from overexpression of *TYRC* with *ARO4* ^*FBR*^ plus *TAL* in different genetic backgrounds. **a** In Aro10^−^ Zwf1^−^ Cdc19^+^ versus Aro10^−^ Zwf1^+^ Cdc19^+^, strain TY1041 versus TY954. **b** In Aro10^−^ Zwf1^−^ Cdc19^+^ with and without methionine added to the growth medium, strain TY1041. **c** In Aro10^−^ Zwf1^−^ Cdc19^+^ versus Aro10^−^ Zwf1^−^ Cdc19^low^ with methionine added to the growth medium, strain TY1031 versus TY1041. Erythrose 4-phosphate, phosphoenolpyruvate and pyruvate were measured intracellularly. Glycerol, acetate and ethanol were measured extracellularly. Values represent an average of three biological replicates and *error bars* represent 95 % confidence intervals. Additional file 7: Figure S7. Estimation of pathway changes in Gibbs free energy for strains created in this study. *ΔG*_*γ*_ values were estimated using the metabolite concentration data obtained in this study combined with values obtained from literature and presented here at 12 h of batch culture on YNB glucose, with supplemented methionine where noted. *Bars* represent the *ΔG*_*γ*_ for each reaction step, while the *lines* depict the cumulative *ΔG*_*γ*_ over the entire pathway. Reactions close to equilibrium are generally less likely to be limited by enzyme level than reactions that are further from equilibrium. Additional file 8: Table S1.
*Saccharomyces cerevisiae* strains used in this study. Additional file 9: Table S2. Primers used in this study. Additional file 10.
**Intracellular and extracellular metabolite concentrations and biomass levels are provided in triplicate for each strain studied over the full experiment time course.**
