# Supplementary figures and images for: Metabolic engineering of a tyrosine-overproducing yeast platform using targeted metabolomics
Source: Microb Cell Fact. 2015 May 28;14:73. doi: 10.1186/s12934-015-0252-2 (PMC4458059; doi:10.1186/s12934-015-0252-2)

A

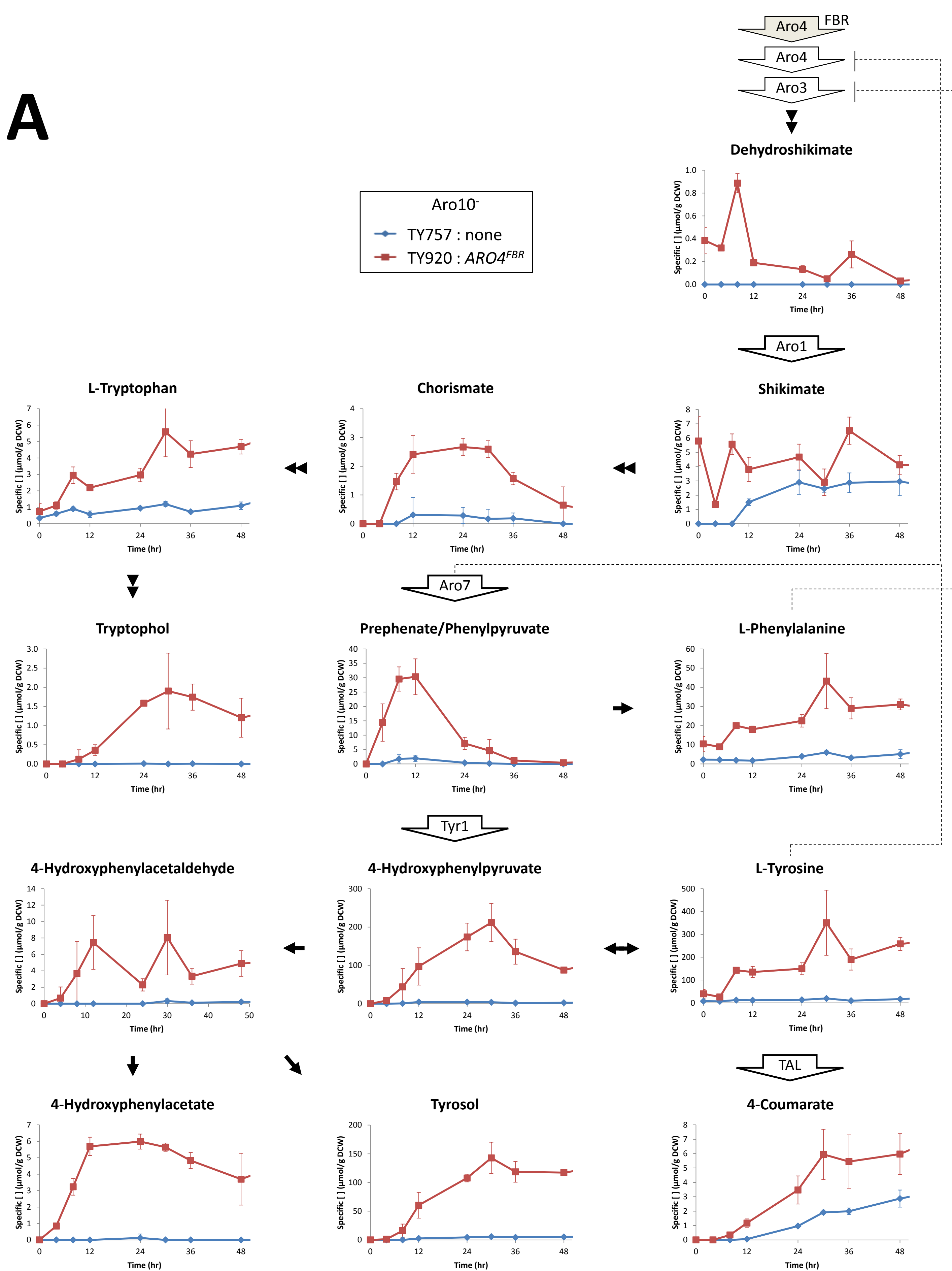

B

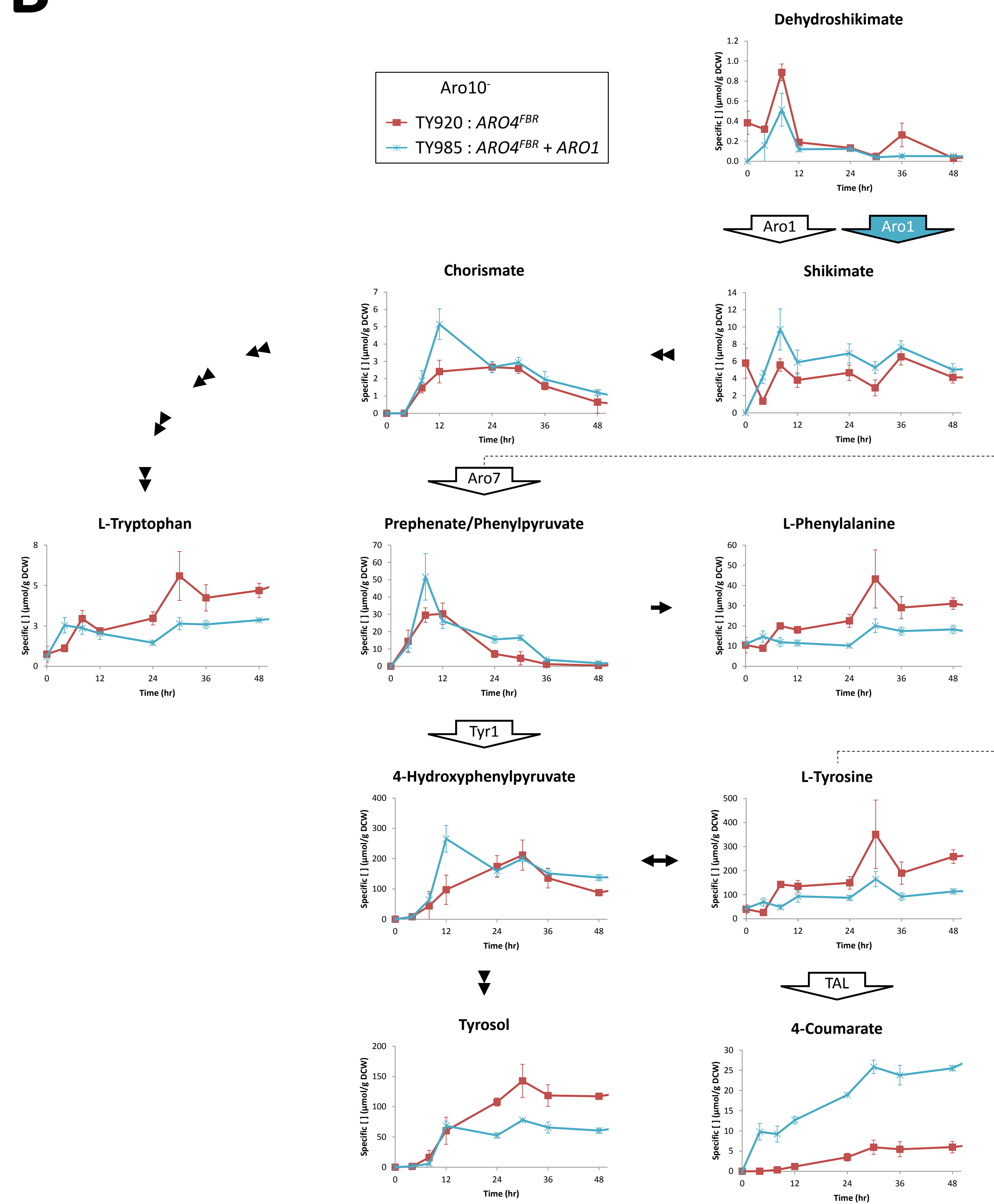

C

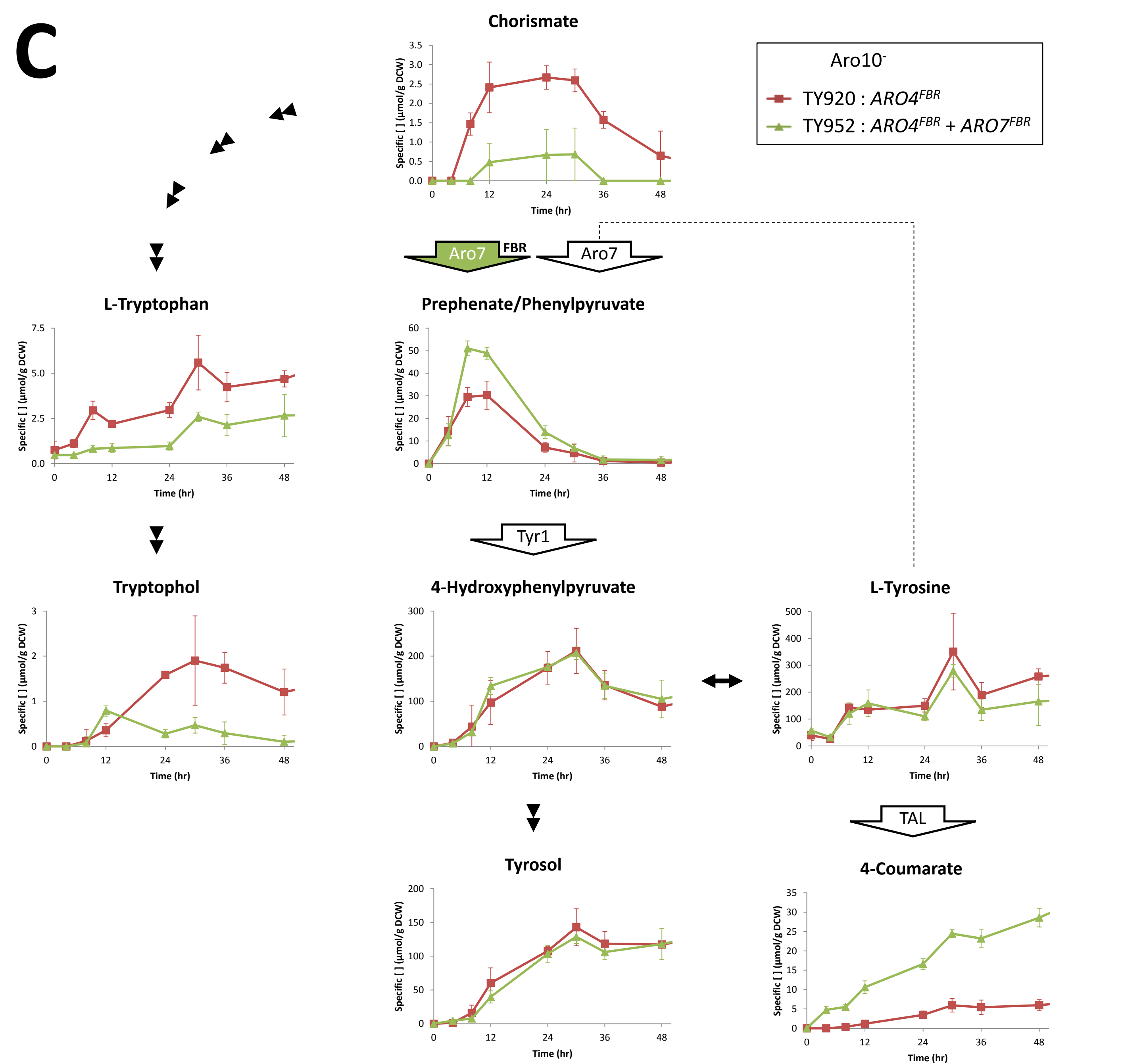

D

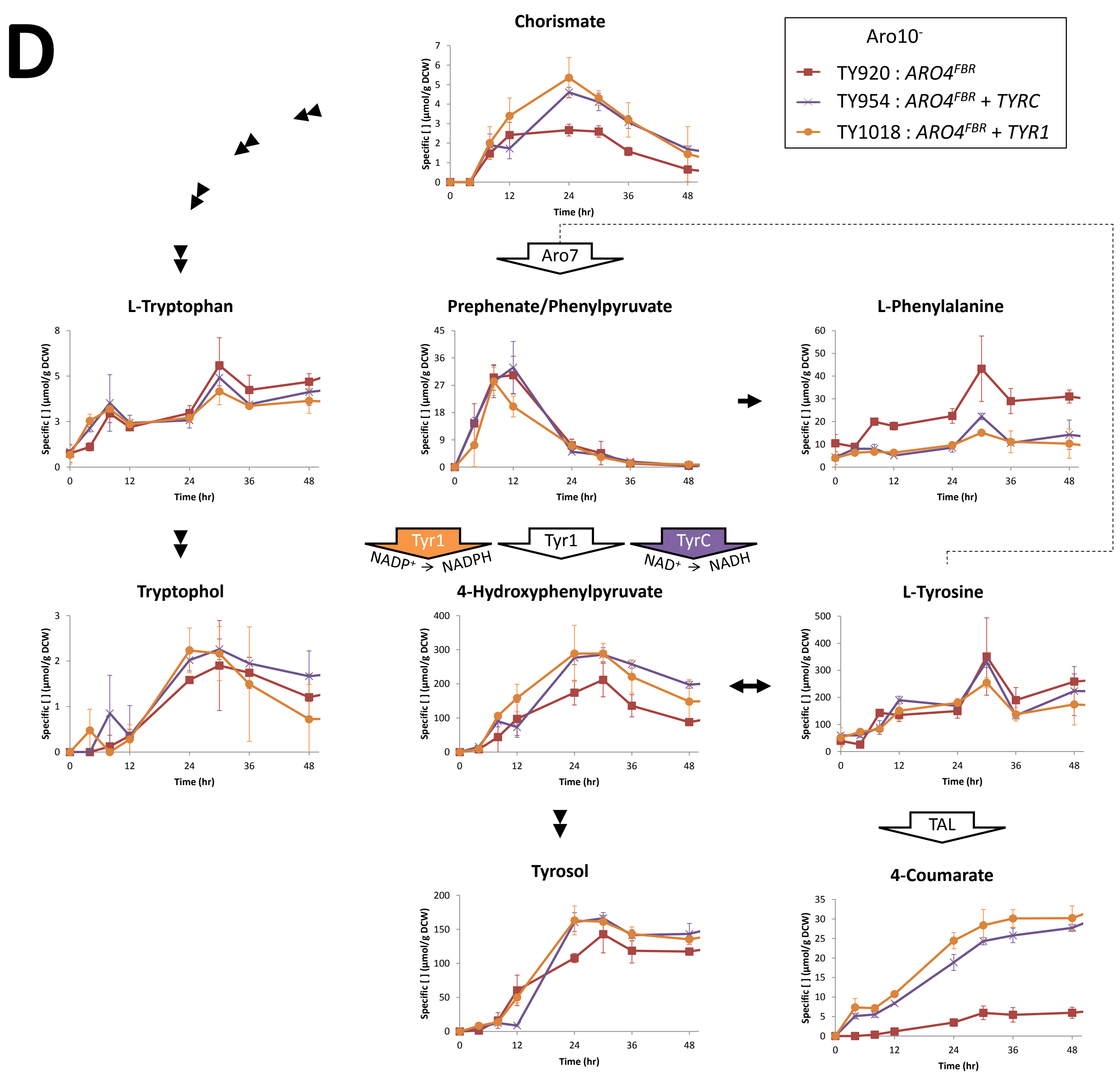

Supplement: Additional file 1: Figure S1. — Aromatic amino acid pathway metabolite profiles from deregulation or overexpression of genes impinging on tyrosine biosynthesis plus TAL in Aro10− CEN.PK. a Overexpression of ARO4 FBR, strain TY920 versus strain TY757. b Overexpression of ARO1 with ARO4 FBR, strain TY985 versus strain TY920. c Overexpression of ARO7 FBR with ARO4 FBR, strain TY952 versus strain TY920. d Overexpression of TYR1 and TYRC with ARO4 FBR, strains TY1018 and TY954 versus strain TY920. Dehydroshikimate, shikimate, L-tryptophan, L-phenylalanine, and L-tyrosine were measured intracellularly. Chorismate, tryptophol, prephenate/phenylpyruvate, 4-hydroxyphenylpyruvate, 4-hydrophenylacetaldehyde, 4-hydroxyphenylacetate, tyrosol, and 4-coumarate were measured extracellularly. Dotted line indicates allosteric feedback inhibition. Values represent an average of three biological replicates and error bars represent 95 % confidence intervals. [file 12934_2015_252_MOESM1_ESM.pdf]

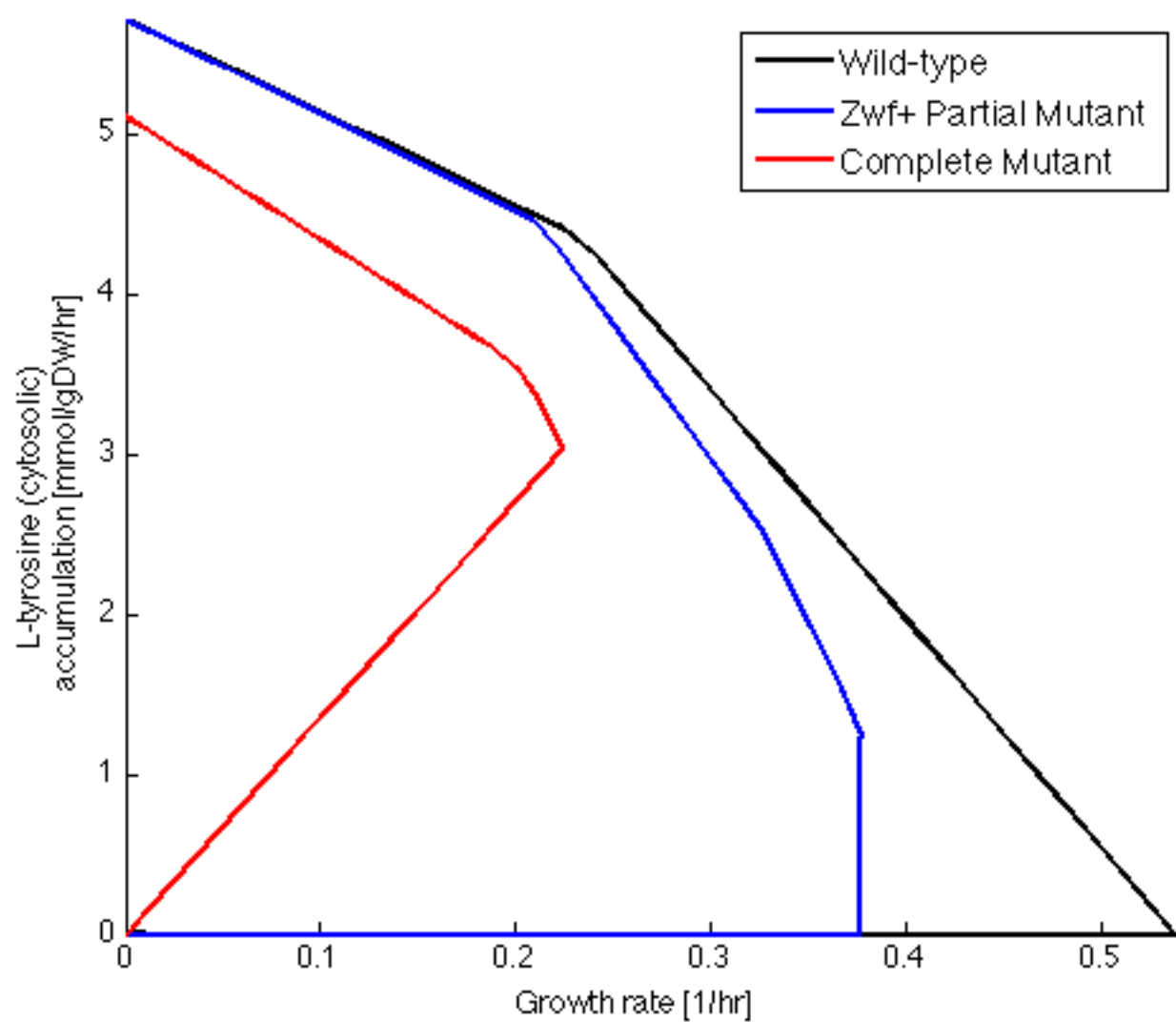

Supplement: Additional file 2: Figure S2. — Tyrosine-overproducing strain design obtained using GDLS. Product-growth envelopes are shown using the iMM904 model with glucose and oxygen uptake set at −10 mmol/g DCW/h. Whereas the wild-type strain (black line) yields no surplus tyrosine at optimal growth, the complete GDLS mutant strain (red line) indicates strong growth coupling and a glucose yield near 60 % theoretical. If the design is implemented with ZWF1 still intact (blue line), tyrosine export is predicted to vary over a range due to alternate optimal solutions in which either tyrosine or phenylalanine can be exported equally. [file 12934_2015_252_MOESM2_ESM.pdf]

A

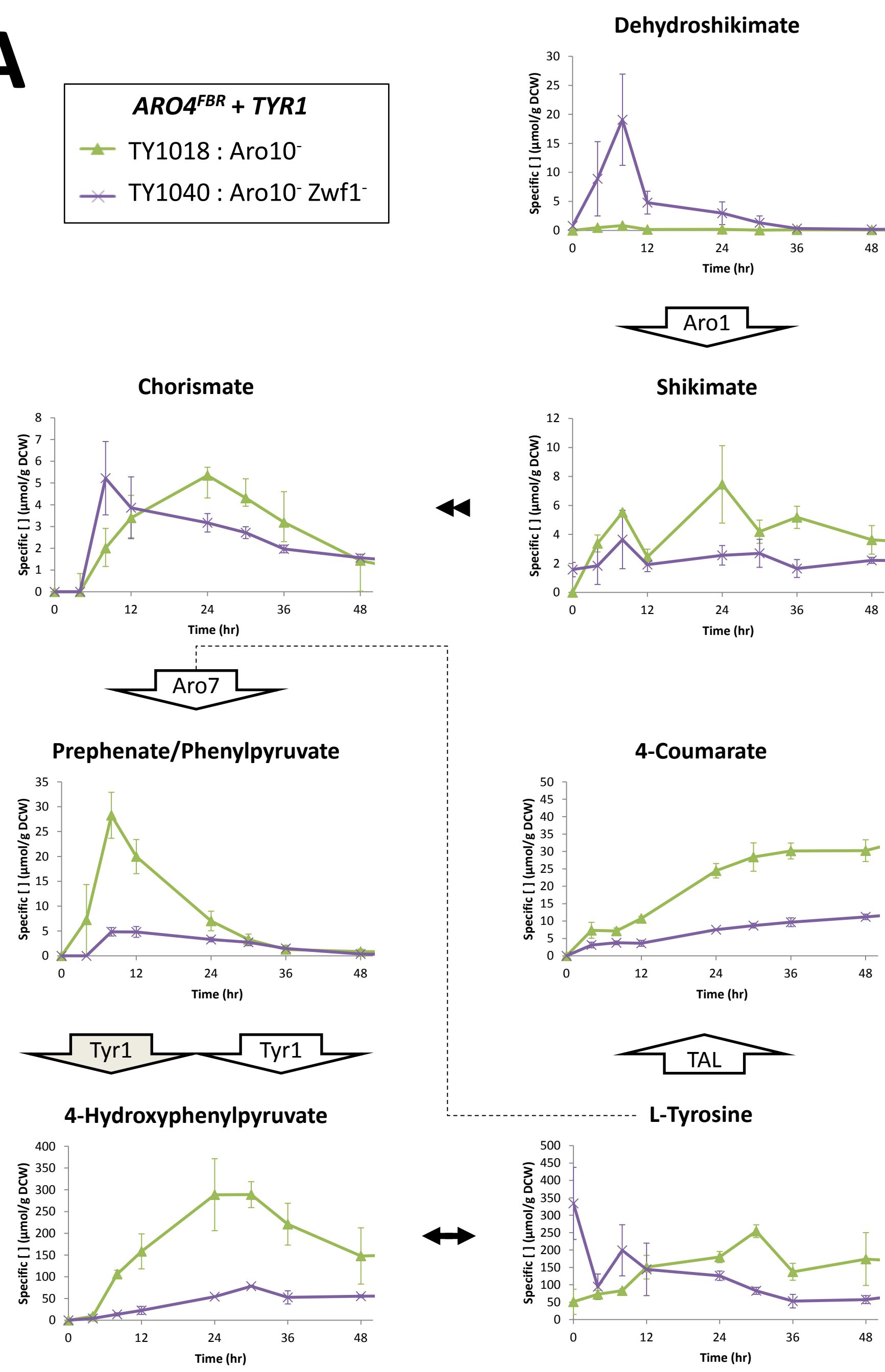

B

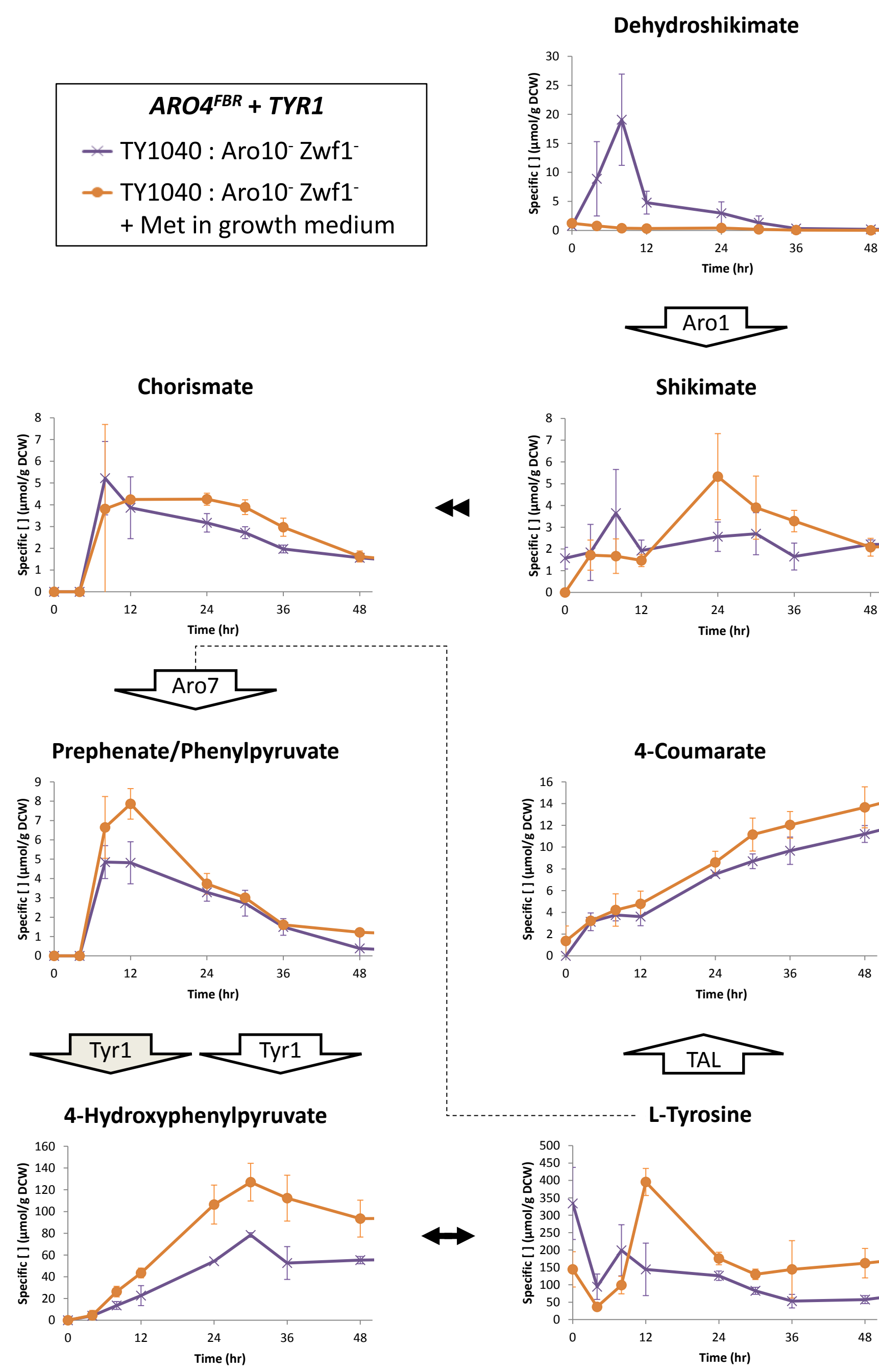

C

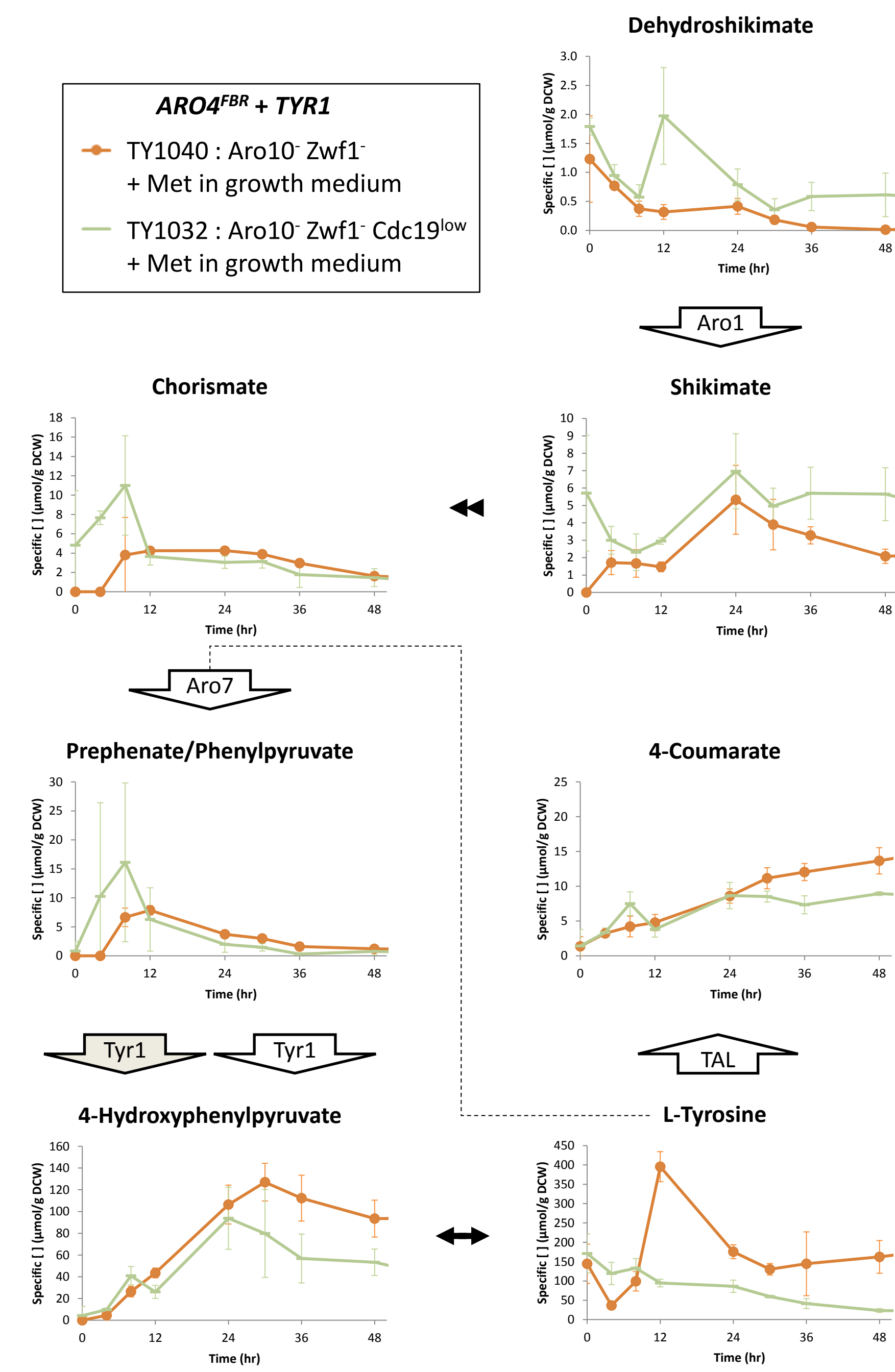

Supplement: Additional file 3: Figure S3. — Aromatic amino acid pathway metabolite profiles from overexpression of TYR1 with ARO4 FBR in different genetic backgrounds. a In Aro10− Zwf1− Cdc19+ versus Aro10− Zwf1+ Cdc19+, strain TY1040 versus TY1018. b In Aro10− Zwf1− Cdc19+ with and without methionine added to the growth medium, strain TY1040. c In Aro10− Zwf1− Cdc19+ versus Aro10− Zwf1− Cdc19low with methionine added to the growth medium, strain TY1032 versus TY1040. Dehydroshikimate, shikimate, and L-tyrosine were measured intracellularly. Chorismate, prephenate/phenylpyruvate, 4-hydroxyphenylpyruvate, and 4-coumarate were measured extracellularly. Values represent an average of three biological replicates and error bars represent 95 % confidence intervals. Dashed line indicates allosteric feedback inhibition. [file 12934_2015_252_MOESM3_ESM.pdf]

A

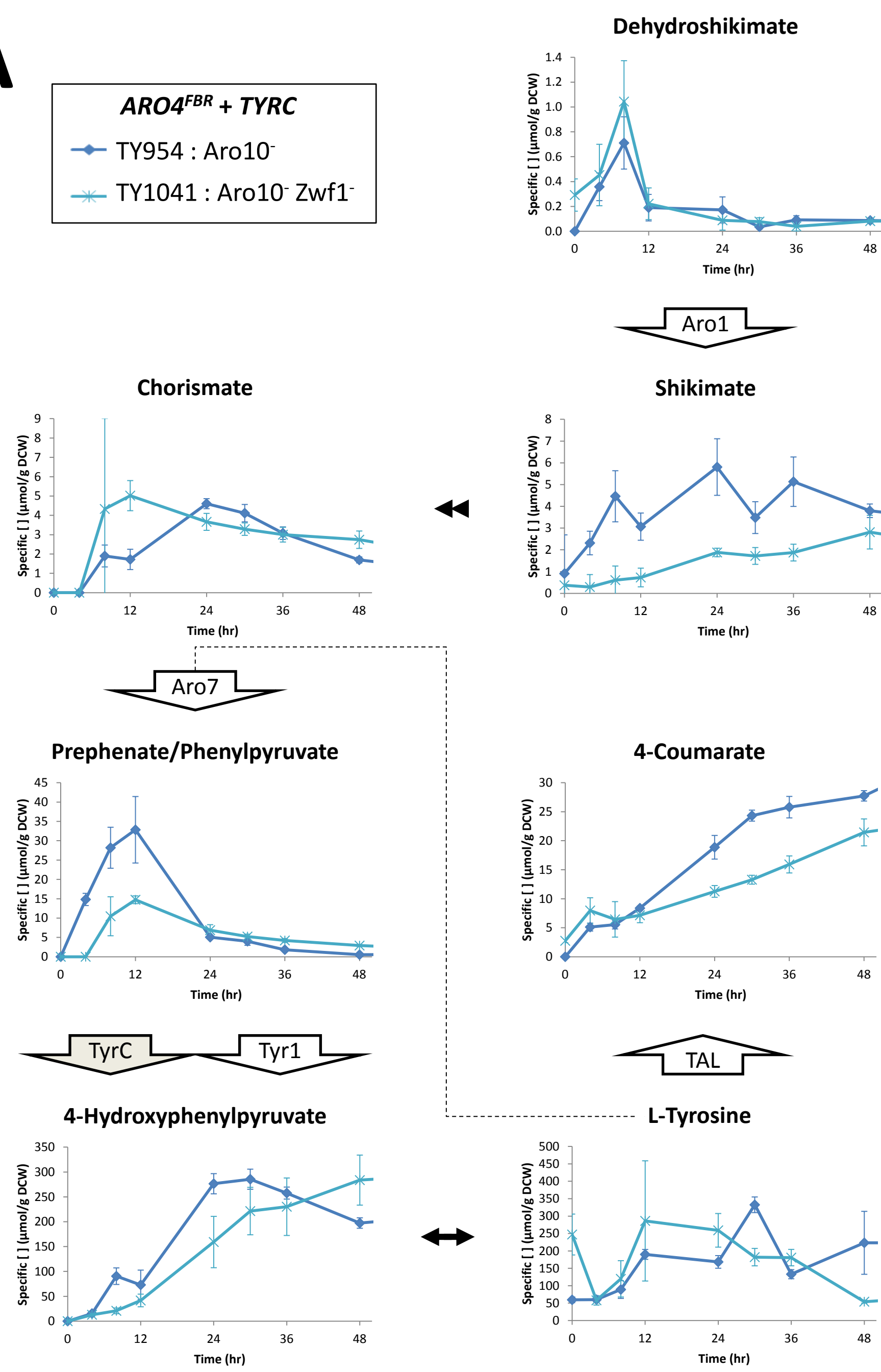

B

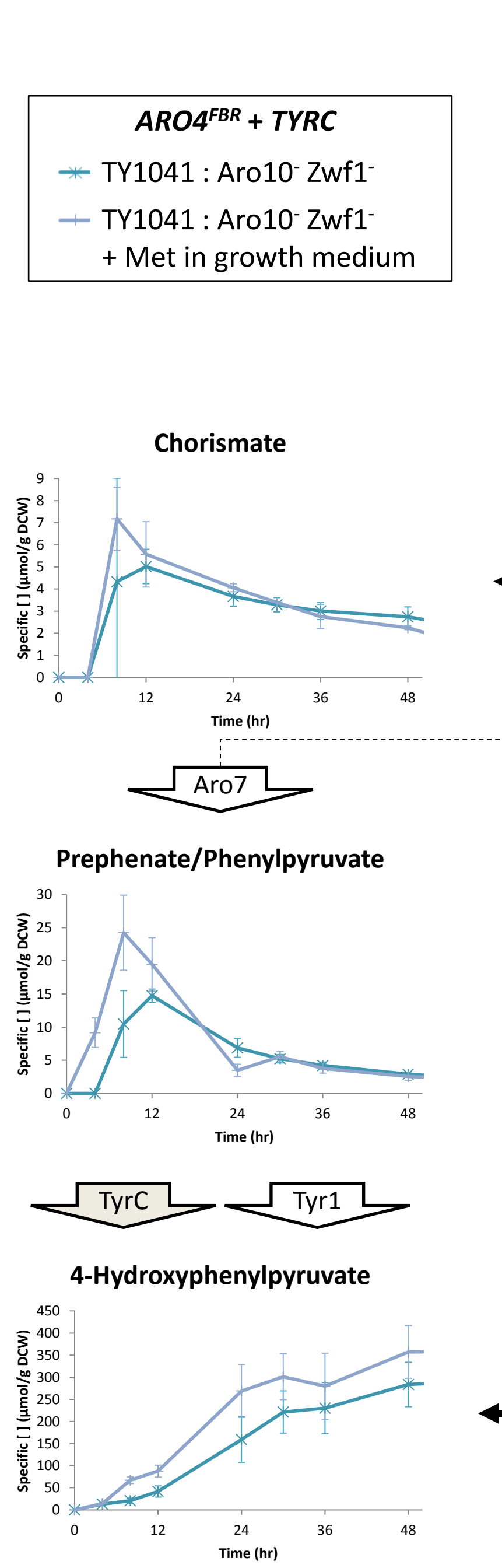

C

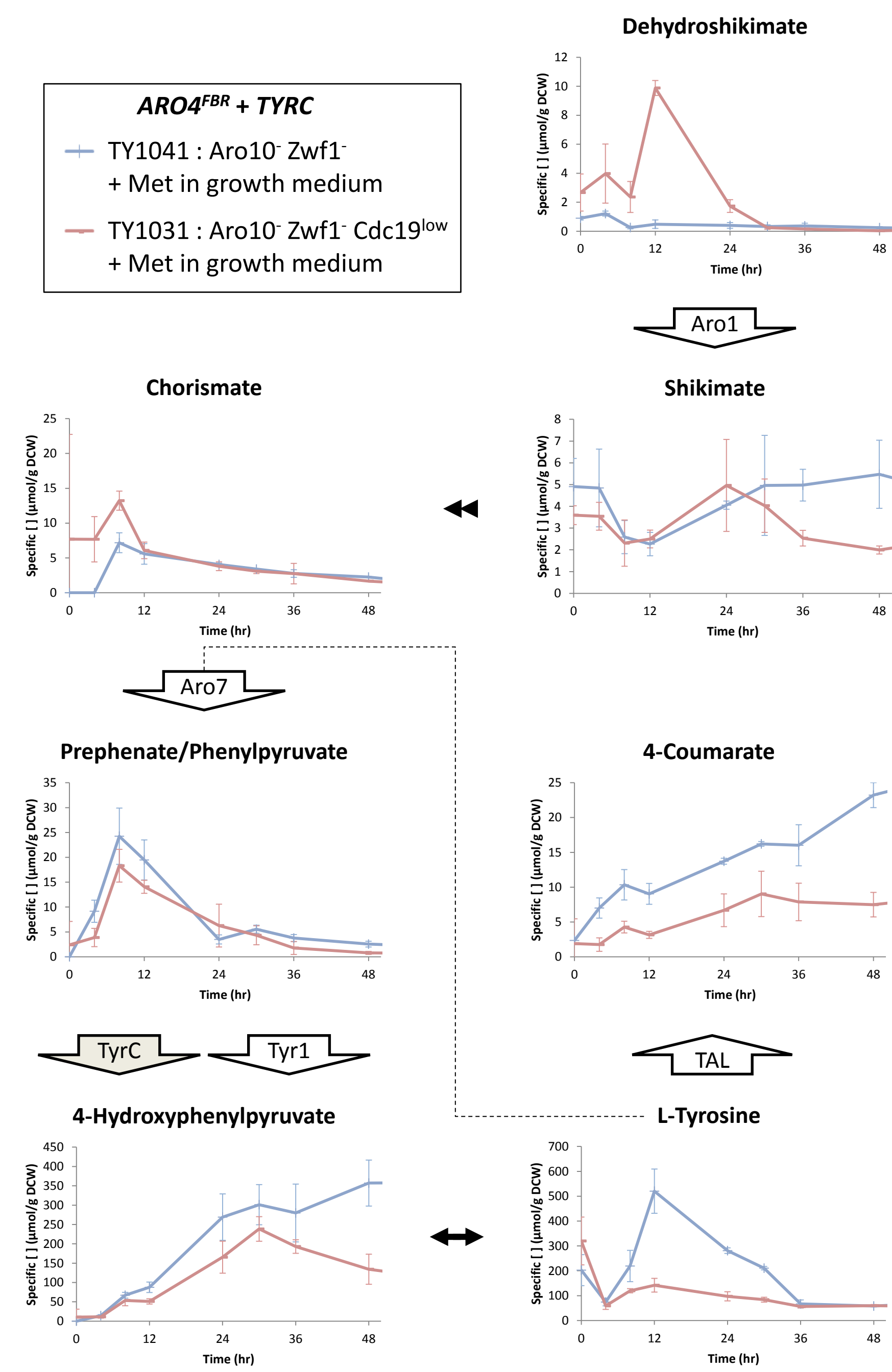

Supplement: Additional file 4: Figure S4. — Aromatic amino acid pathway metabolite profiles from overexpression of TYRC with ARO4 FBR plus TAL in different genetic backgrounds. a In Aro10− Zwf1− Cdc19+ versus Aro10− Zwf1+ Cdc19+, strain TY1041 versus TY954. b In Aro10− Zwf1− Cdc19+ with and without methionine added to the growth medium, strain TY1041. c In Aro10− Zwf1− Cdc19+ versus Aro10− Zwf1− Cdc19low with methionine added to the growth medium, strain TY1031 versus TY1041. Dehydroshikimate, shikimate, and L-tyrosine were measured intracellularly. Chorismate, prephenate/phenylpyruvate, 4-hydroxyphenylpyruvate, and 4-coumarate were measured extracellularly. Values represent an average of three biological replicates and error bars represent 95 % confidence intervals. Dashed line indicates allosteric feedback inhibition. [file 12934_2015_252_MOESM4_ESM.pdf]

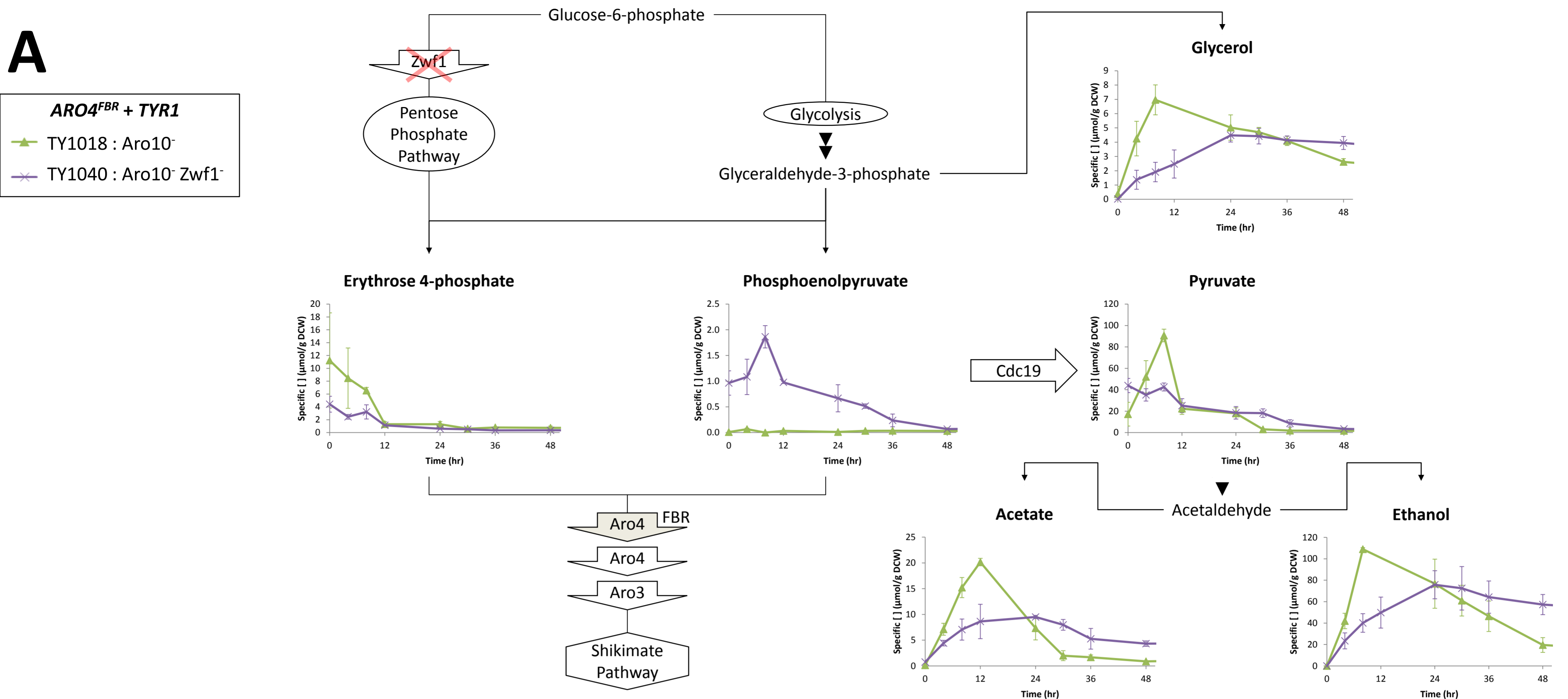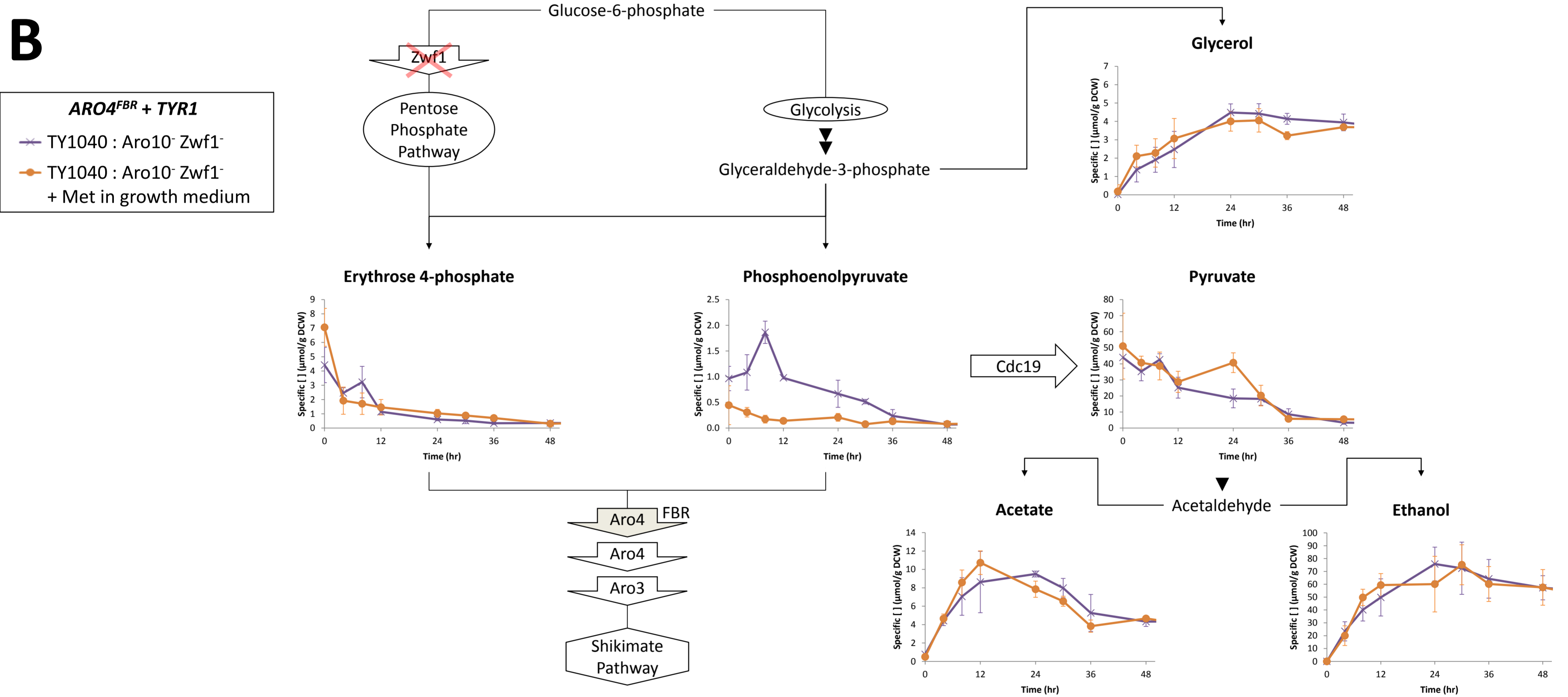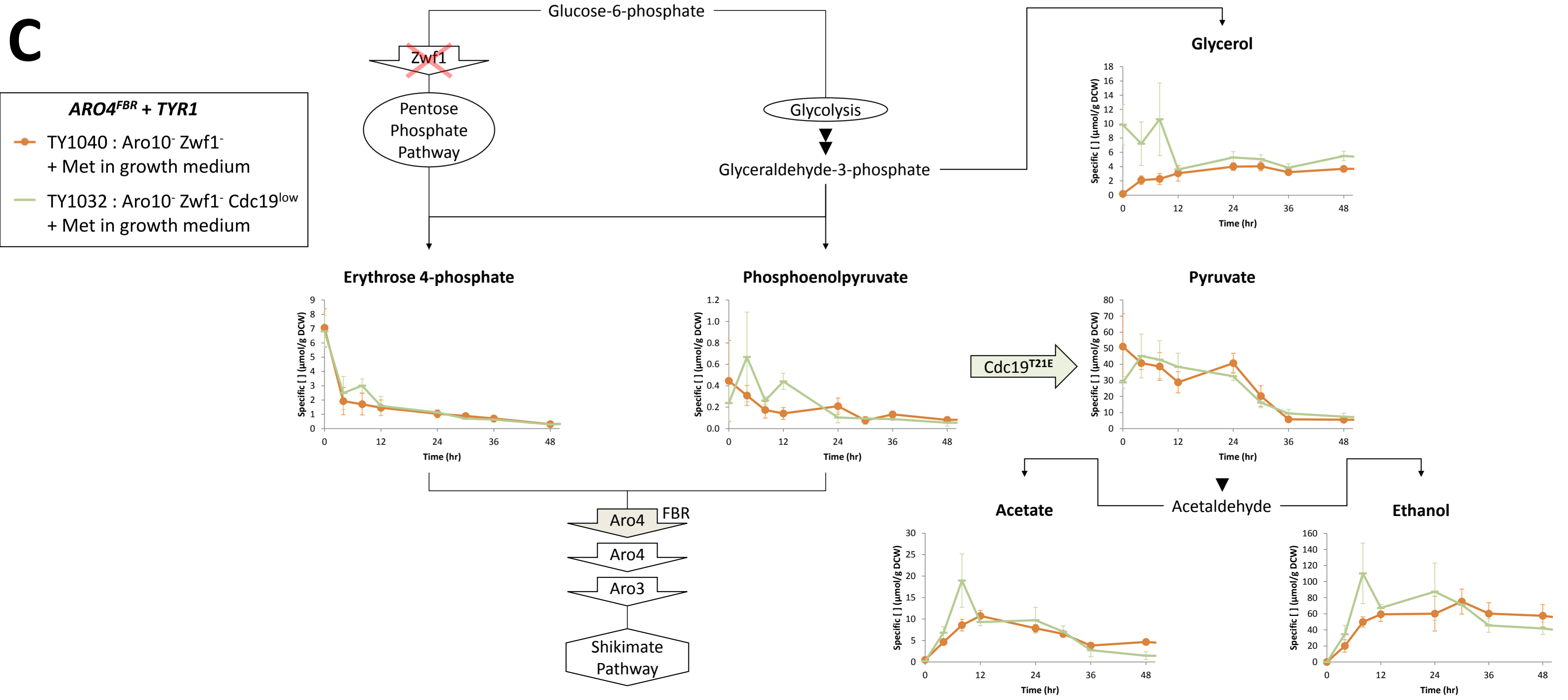

Supplement: Additional file 5: Figure S5. — Shikimate pathway precursors and overflow metabolites from overexpression of TYR1 with ARO4 FBR plus TAL in different genetic backgrounds. a In Aro10− Zwf1− Cdc19+ versus Aro10− Zwf1+ Cdc19+, strain TY1040 versus TY1018. b In Aro10− Zwf1− Cdc19+ with and without methionine added to the growth medium, strain TY1040. c In Aro10− Zwf1− Cdc19+ versus Aro10− Zwf1− Cdc19low with methionine added to the growth medium, strain TY1032 versus TY1040. Erythrose 4-phosphate, phosphoenolpyruvate and pyruvate were measured intracellularly. Glycerol, acetate and ethanol were measured extracellularly. Values represent an average of three biological replicates and error bars represent 95 % confidence intervals. [file 12934_2015_252_MOESM5_ESM.pdf]

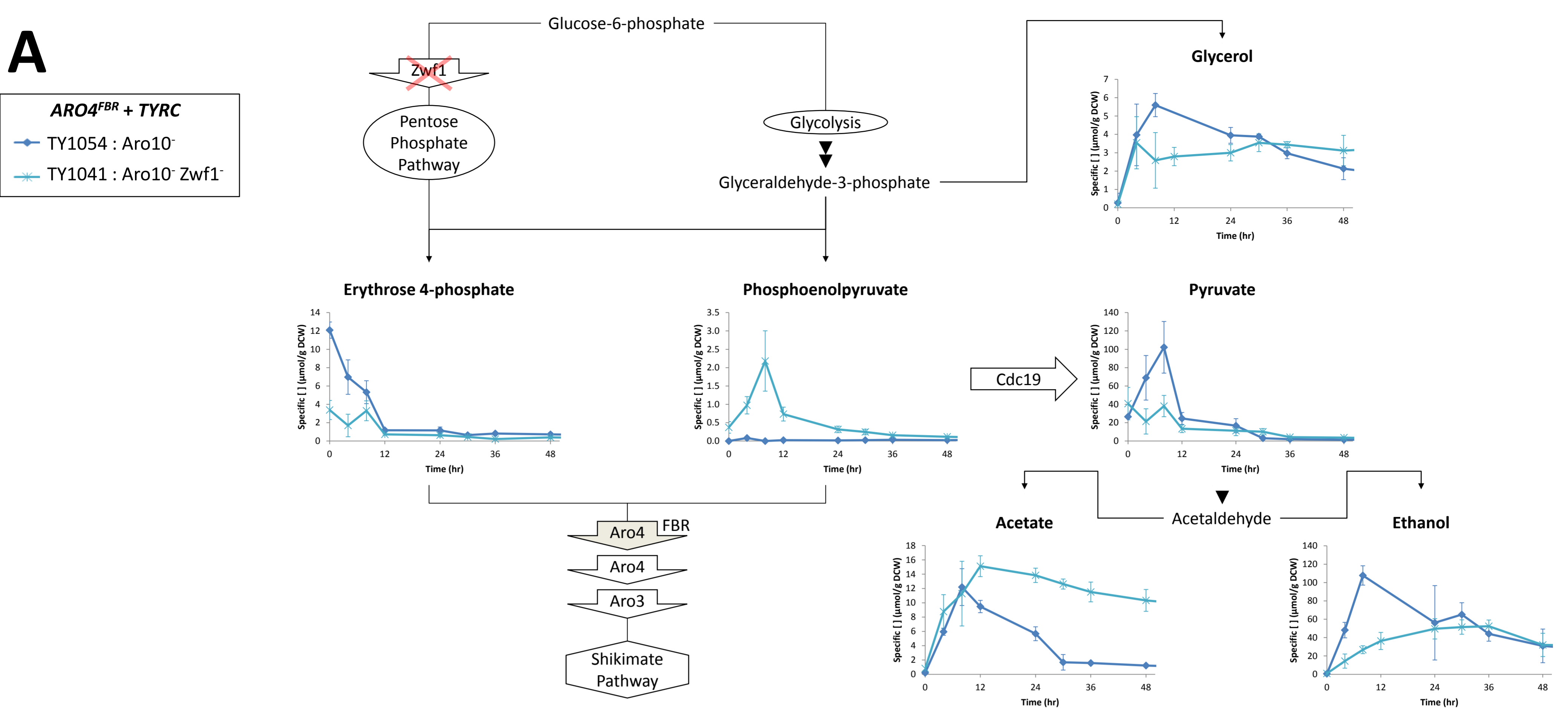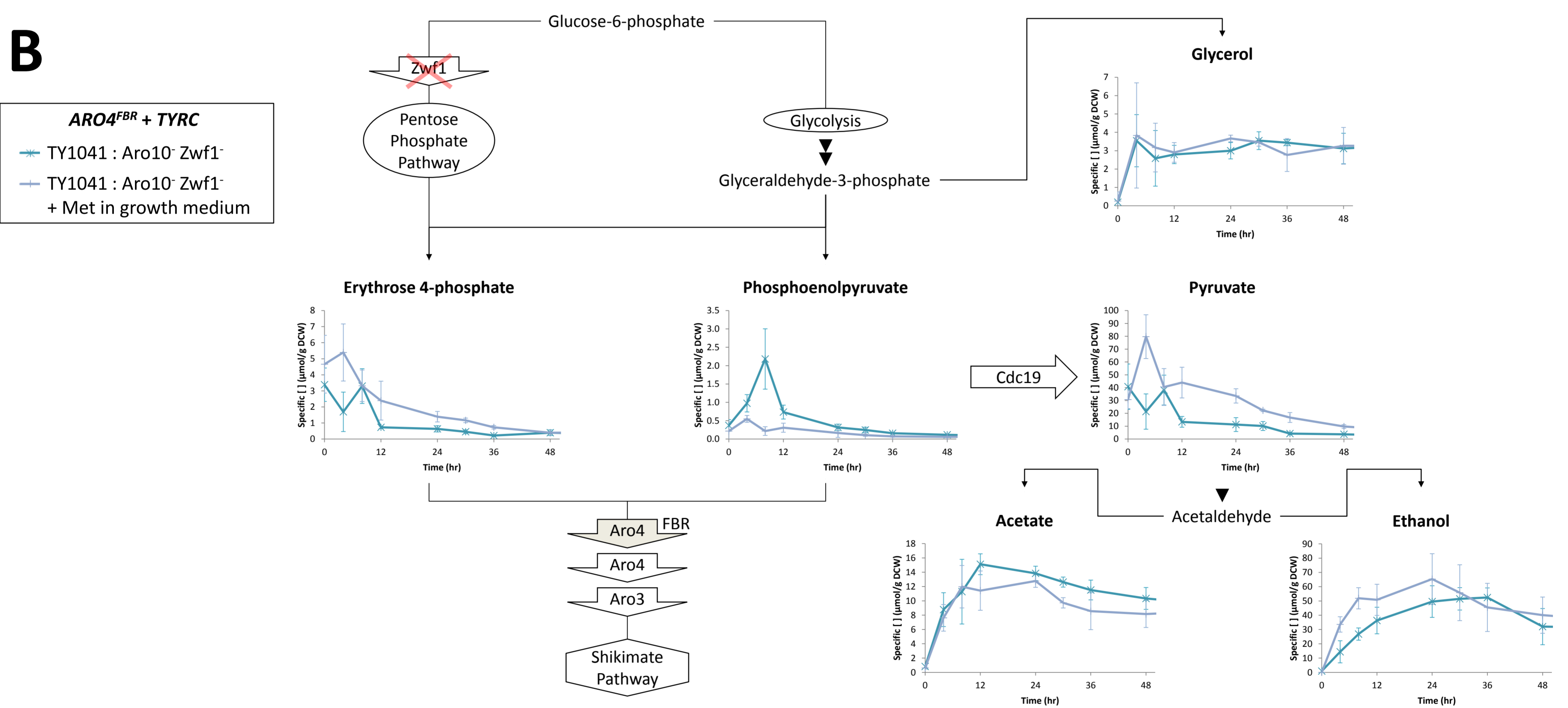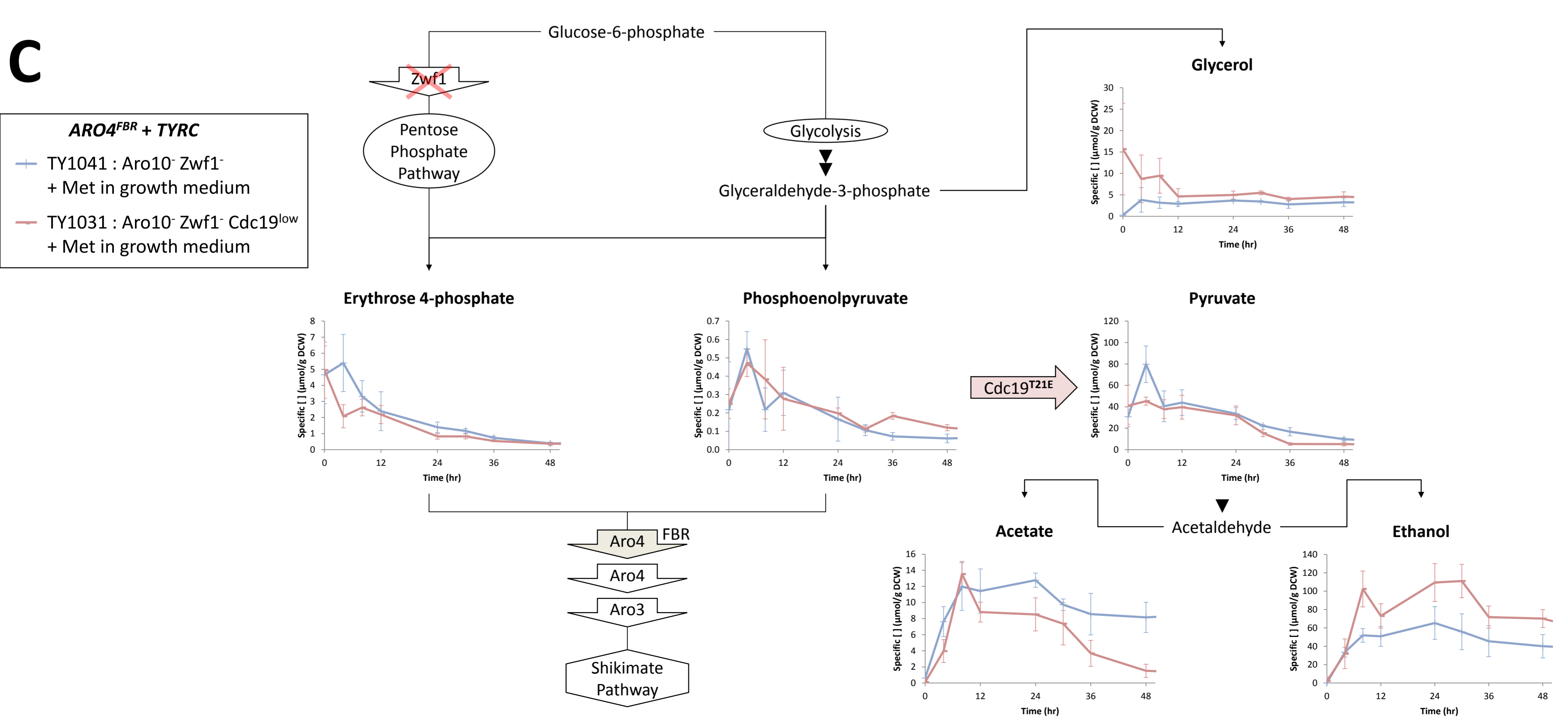

Supplement: Additional file 6: Figure S6. — Shikimate pathway precursors and overflow metabolites from overexpression of TYRC with ARO4 FBR plus TAL in different genetic backgrounds. a In Aro10− Zwf1− Cdc19+ versus Aro10− Zwf1+ Cdc19+, strain TY1041 versus TY954. b In Aro10− Zwf1− Cdc19+ with and without methionine added to the growth medium, strain TY1041. c In Aro10− Zwf1− Cdc19+ versus Aro10− Zwf1− Cdc19low with methionine added to the growth medium, strain TY1031 versus TY1041. Erythrose 4-phosphate, phosphoenolpyruvate and pyruvate were measured intracellularly. Glycerol, acetate and ethanol were measured extracellularly. Values represent an average of three biological replicates and error bars represent 95 % confidence intervals. [file 12934_2015_252_MOESM6_ESM.pdf]

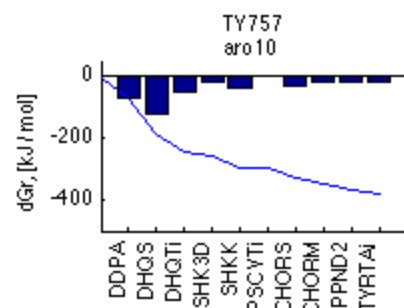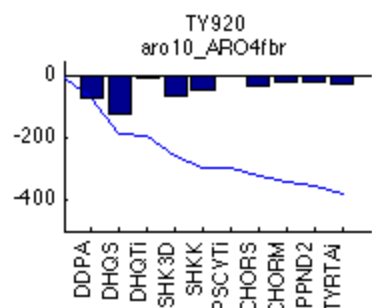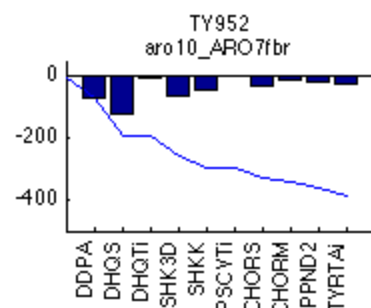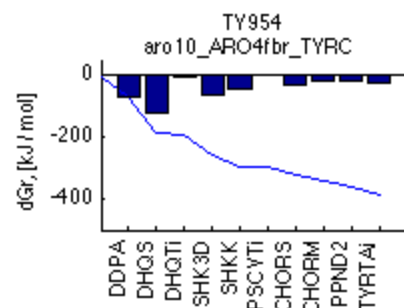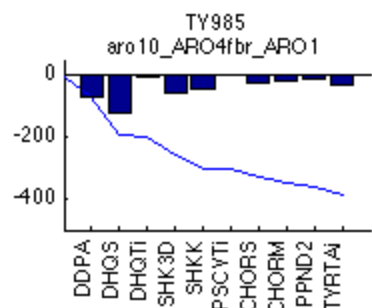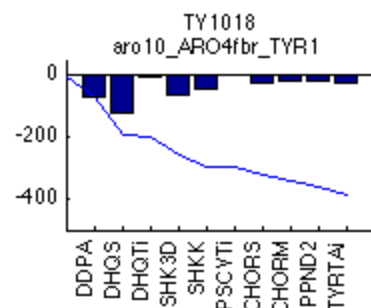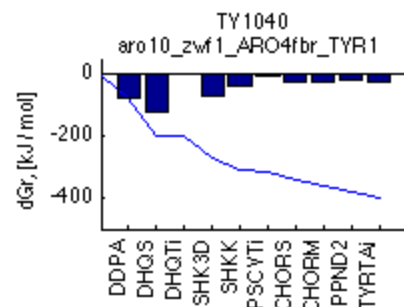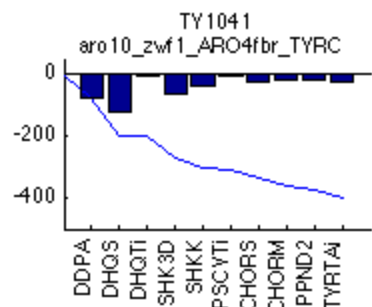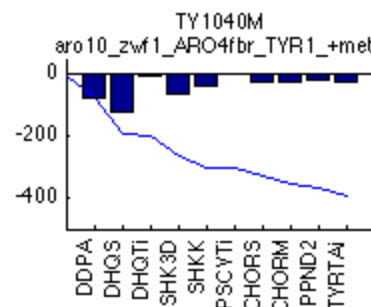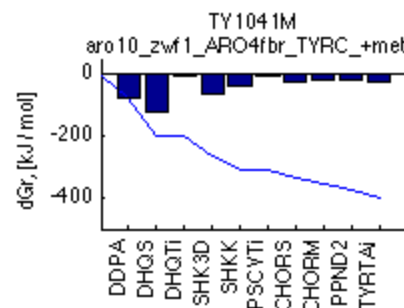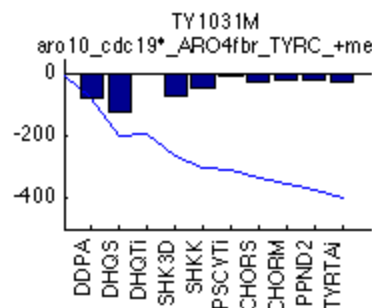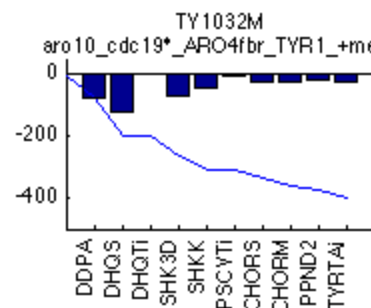

Supplement: Additional file 7: Figure S7. — Estimation of pathway changes in Gibbs free energy for strains created in this study. ΔG γ values were estimated using the metabolite concentration data obtained in this study combined with values obtained from literature and presented here at 12 h of batch culture on YNB glucose, with supplemented methionine where noted. Bars represent the ΔG γ for each reaction step, while the lines depict the cumulative ΔG γ over the entire pathway. Reactions close to equilibrium are generally less likely to be limited by enzyme level than reactions that are further from equilibrium. [file 12934_2015_252_MOESM7_ESM.pdf]
