# Supplementary material for: Metabolic engineering of a tyrosine-overproducing yeast platform using targeted metabolomics
Source: Microb Cell Fact. 2015 May 28;14:73. doi: 10.1186/s12934-015-0252-2 (PMC4458059; doi:10.1186/s12934-015-0252-2)
Supplement: Additional file 8: Table S1. — Saccharomyces cerevisiae strains used in this study. [file 12934_2015_252_MOESM8_ESM.docx]

**Table S1. *Saccharomyces cerevisiae* strains used in this study**

| **Strain** | **Genotype: chromosome**  **Genotype: plasmid** | **Reference** |
| --- | --- | --- |
| CEN.PK111-61A | *MATα ura3 leu2 his3* | [30] |
| CEN.PK111-5B | *MATa ura3 leu2 his3* | [30] |
| JR201 | *MATa ura3 leu2 his3 trp1 cdc19Δ::cdc19^T21E^*-*kanMX* | [27] |
| H703 | *MATα ura3 leu2 his3 aro10Δ*::*hphNT1* | This study |
| H712 | *MATa ura3 leu2 his3 zwf1Δ*::*hphNT1* | This study |
| H749 | *MATα ura3 leu2 his3* *cdc19Δ::cdc19^T21E^*-*kanMX* | This study |
| H837 | *MATα ura3 leu2 his3 aro10-Δ* | This study |
| H1045 | *MATα ura3 leu2 his3* *aro10Δ*::*hphNT1* *cdc19Δ::cdc19^T21E^*-*kanMX* | This study |
| H919 | *MATa ura3 leu2 his3* *aro10Δ*::*hphNT1* *zwf1Δ*::*hphNT1* | This study |
| H876 | *MATα ura3 leu2 his3* *aro10Δ*::*hphNT1* *cdc19Δ::cdc19^T21E^*-*kanMX* *zwf1Δ*::*hphNT1* | This study |
| TY757 | *MATα ura3 leu2 his3 aro10-Δ*  pTY350 (*2µ LEU2 PMA1_pr_-TAL*) pTY338 (*CEN.ARS URA3*) pTY51 (*CEN.ARS HIS3*) | This study |
| TY920 | *MATα ura3 leu2 his3 aro10-Δ*  pTY350 (*2µ LEU2 PMA1_pr_-TAL*) pTY798 (*CEN.ARS URA3 FBA1_pr_-ARO4^K229L^*) pTY51 (*CEN.ARS HIS3*) | This study |
| TY985 | *MATα ura3 leu2 his3 aro10-Δ*  pTY350 (*2µ LEU2 PMA1_pr_-TAL*) pTY798 (*CEN.ARS URA3 FBA1_pr_-ARO4^K229L^*) pTY502 (*CEN.ARS HIS3 PYK1_pr_-ARO1*) | This study |
| TY952 | *MATα ura3 leu2 his3 aro10-Δ*  pTY350 (*2µ LEU2 PMA1_pr_-TAL*) pTY798 (*CEN.ARS URA3 FBA1_pr_-ARO4^K229L^*) pTY688 (*CEN.ARS HIS3 PDC1_pr_-ARO7^G141S^*) | This study |
| TY954 | *MATα ura3 leu2 his3 aro10-Δ*  pTY350 (*2µ LEU2 PMA1_pr_-TAL*) pTY798 (*CEN.ARS URA3 FBA1_pr_-ARO4^K229L^*) pTY500 (*CEN.ARS HIS3 TDH3_pr_-TYRC*) | This study |
| TY1018 | *MATα ura3 leu2 his3 aro10-Δ*  pTY350 (*2µ LEU2 PMA1_pr_-TAL*) pTY798 (*CEN.ARS URA3 FBA1_pr_-ARO4^K229L^*) pTY1035 (*CEN.ARS HIS3 TEF1_pr_-TYR1*) | This study |
| TY1041 | *MATa ura3 leu2 his3* *aro10Δ*::*kanMX* *zwf1Δ*::*hphNT1*  pTY350 (*2µ LEU2 PMA1_pr_-TAL*) pTY798 (*CEN.ARS URA3 FBA1_pr_-ARO4^K229L^*) pTY500 (*CEN.ARS HIS3 TDH3_pr_-TYRC*) | This study |
| TY1040 | *MATa ura3 leu2 his3* *aro10Δ*::*kanMX* *zwf1Δ*::*hphNT1*  pTY350 (*2µ LEU2 PMA1_pr_-TAL*) pTY798 (*CEN.ARS URA3 FBA1_pr_-ARO4^K229L^*) pTY1035 (*CEN.ARS HIS3 TEF1_pr_-TYR1*) | This study |
| TY1031 | *MATα ura3 leu2 his3* *aro10Δ*::*hphNT1* *cdc19Δ::cdc19^T21E^*-*kanMX* *zwf1Δ*::*hphNT1*  pTY350 (*2µ LEU2 PMA1_pr_-TAL*) pTY798 (*CEN.ARS URA3 FBA1_pr_-ARO4^K229L^*) pTY500 (*CEN.ARS HIS3 TDH3_pr_-TYRC*) | This study |
| TY1032 | *MATα ura3 leu2 his3* *aro10Δ*::*hphNT1* *cdc19Δ::cdc19^T21E^*-*kanMX* *zwf1Δ*::*hphNT1*  pTY350 (*2µ LEU2 PMA1_pr_-TAL*) pTY798 (*CEN.ARS URA3 FBA1_pr_-ARO4^K229L^*) pTY1035 (*CEN.ARS HIS3 TEF1_pr_-TYR1*) | This study |
