# Supplementary material for: Metabolic engineering of a tyrosine-overproducing yeast platform using targeted metabolomics
Source: Microb Cell Fact. 2015 May 28;14:73. doi: 10.1186/s12934-015-0252-2 (PMC4458059; doi:10.1186/s12934-015-0252-2)
Supplement: Additional file 9: Table S2. — Primers used in this study. [file 12934_2015_252_MOESM9_ESM.docx]

Table S2. Primers used in this study

| **Used for** | **Name 5'-3'** | **Sequence 5'-3' (**Kozak**,** mutation**)** |
| --- | --- | --- |
| **Gene assemblies** | | |
| pYES2-prom.PMA1-TAL | | |
|  | PMA1p-TAL_F | AGATAAGAAAGATTTAATTATCAAAAAAACAATGTTGGCCATGTC |
|  | PMA1p-TAL_R | GGTGGTGACATGGCCAACATTGTTTTTTTGATAATTAAATCTTTC |
|  |  |  |
| pYES2-ARO4fbr | | |
|  | Aro4mutfor | CATTTCATGGGTGTTACTTTGCATGGTGTT |
|  | Aro4mutrev | TAGCAGCAACACCATGCAAAGTAACACCCA |
| prom.FBA1-ARO4fbr-term.TDH2 | | |
|  | H506-C1 F | TAACCCTCACTAAAGGGAACAAAAGCTGGAGCTCGTTTAAACGGCGCGCCGAGACTGCAGCATTACTTTGAGAAG |
|  | C1-prom.FBA1 F | GAGACTGCAGCATTACTTTGAGAAGATCCAACTGGCACCGCTGGC |
|  | Aro4fbr-Kzk-prom.FBA1 R | CAGCGAACATTGGAGATTCACTCATtgttTTATGTATTACTTGGTTATGGTTATATATGACAAA |
|  | prom.FBA1-Kzk-Aro4fbr F | CCATAACCAAGTAATACATAAaacaATGAGTGAATCTCCAATGTTCGCTG |
|  | term.TDH2-Aro4fbr R | AGTAACTTAAGGAGTTAAATCTATTTCTTGTTAACTTCTCTTCTTTGTCT |
|  | Aro4fbr-term.TDH2 F | AGACAAAGAAGAGAAGTTAACAAGAAATAGATTTAACTCCTTAAGTTACTTTAATGATTTAGTTTTTATT |
|  | C6-term.TDH2 R | ACAACTCATGGTGATGTGATTGCCGCGAAAAGCCAATTAGTGTGATAC |
|  | H506'-C6 R | ATAACTTCGTATAATGTATGCTATACGAAGTTATTAGGTACCGCGGCCGCACAACTCATGGTGATGTGATTGCC |
| prom.PDC1-ARO7fbr-term.ADH2 | | |
|  | H506-C1 F | TAACCCTCACTAAAGGGAACAAAAGCTGGAGCTCGTTTAAACGGCGCGCCGAGACTGCAGCATTACTTTGAGAAG |
|  | C1-prom.PDC1 F | GAGACTGCAGCATTACTTTGAGAAGACATGCGACTGGGTGAGCATATG |
|  | ARO7fbr-Kzk-prom.PDC1 R | TGGTTTTGTGAAATCCATtgttTTGATTTGACTGTGTTATTTTGCGTGAG |
|  | prom.PDC1-Kzk-ARO7fbr F | CTCACGCAAAATAACACAGTCAAATCAAaacaATGGATTTCACAAAACCAGAAACTGTTTTAAA |
|  | term.ADH2-ARO7fbr R | ATCGTAAAGACATAAGAGATCCGCTTACTCTTCCAACCTTCTTAGCAAGT |
|  | ARO7_introFBR_bottomHalf F | TGGTGATGATAAGAATAACTTCGGTTCTGTTGCCACTAGAGATATAGAAT |
|  | ARO7_introFBR_topHalf R | ATTCTATATCTCTAGTGGCAACAGAACCGAAGTTATTCTTATCATCACCA |
|  | ARO7fbr-term.ADH2 F | ACTTGCTAAGAAGGTTGGAAGAGTAAGCGGATCTCTTATGTCTTTACGATTTA |
|  | C6-term.ADH2 R | ACAACTCATGGTGATGTGATTGCCTAGAATTATATAACTTGATGAGATGAGATGAG |
|  | H506'-C6 R | ATAACTTCGTATAATGTATGCTATACGAAGTTATTAGGTACCGCGGCCGCACAACTCATGGTGATGTGATTGCC |
| prom.TEF1-TYR1-term.FBA1 | | |
|  | H506-C1 F | TAACCCTCACTAAAGGGAACAAAAGCTGGAGCTCGTTTAAACGGCGCGCCGAGACTGCAGCATTACTTTGAGAAG |
|  | TYR1-TEF1f_R | TCCATTGCTCAATCTTATCCTCTGATACCATTTTGTAATTAAAACTTAGATTAGATTGC |
|  | TEF1p-TYR1_FII | AATCTAATCTAAGTTTTAATTACAAAATGGTATCAGAGGATAAGATTGAGCAATGGAAAG |
|  | FBA1t-TYR1_RII | ACTATATCAATTAATTTGAATTAACTTATGTATTTCTTTTTTCAGCGGCCGAACGGTCAC |
|  | TYR1-FBA1t_F | AGTGACCGTTCGGCCGCTGAAAAAAGAAATACATAAGTTAATTCAAATTAATTGATATAG |
|  | H506'-C6 R | ATAACTTCGTATAATGTATGCTATACGAAGTTATTAGGTACCGCGGCCGCACAACTCATGGTGATGTGATTGCC |
| prom.TDH3-TYRC-term.ENO2 | | |
|  | H506-C1 F | TAACCCTCACTAAAGGGAACAAAAGCTGGAGCTCGTTTAAACGGCGCGCCGAGACTGCAGCATTACTTTGAGAAG |
|  | C1-prom.TDH3 F | GAGACTGCAGCATTACTTTGAGAAGTCGAGTTTATCATTATCAATAC |
|  | TyrC-Kzk-prom.TDH3 R | AATGGCAATATGCTTAAAGACGGTCATtgttTTCGAAACTAAGTTCTTGGTGTTTTAAAACT |
|  | prom.TDH3-Kzk-TyrC F | CCAAGAACTTAGTTTCGAAaacaATGACCGTCTTTAAGCATATTGCCATT |
|  | term.ENO2-TyrC R | GACTAATAATTCTTAGTTAAAAGCACTTTAAGGGCGAATATCGTGGTCTG |
|  | TyrC-term.ENO2 F | CAGACCACGATATTCGCCCTTAAAGTGCTTTTAACTAAGAATTATTAGTCTTTTCTG |
|  | C6-term.ENO2 R | ACAACTCATGGTGATGTGATTGCCAGGTATCATCTCCATCTCCCATATG |
|  | H506'-C6 R | ATAACTTCGTATAATGTATGCTATACGAAGTTATTAGGTACCGCGGCCGCACAACTCATGGTGATGTGATTGCC |
| prom.PYK1-ARO1-term.PGI1 | | |
|  | H506-C1 F | TAACCCTCACTAAAGGGAACAAAAGCTGGAGCTCGTTTAAACGGCGCGCCGAGACTGCAGCATTACTTTGAGAAG |
|  | C1-prom.PYK1 F | GAGACTGCAGCATTACTTTGAGAAGAATGCTACTATTTTGGAGATTAATCTCAG |
|  | ARO1-Kzk-prom.PYK1 R | GGCTAACTGCACCATtgttTGATGTTTTATTTGTTTTGATTGGTGTCTTG |
|  | prom.PYK1-Kzk-ARO1 F | CAAGACACCAATCAAAACAAATAAAACATCAaacaATGGTGCAGTTAGCCAAAGTCC |
|  | term.PGI1-ARO1 R | GTATATATTTAAGAGCGATTTGTTCTACTCTTTCGTAACGGCATCAAAAA |
|  | ARO1-term.PGI1 F | TTTTTGATGCCGTTACGAAAGAGTAGAACAAATCGCTCTTAAATATATACCTAAAGAAC |
| **Chromosomal deletion & mutation cassettes** | | |
|  | Aro10 Del F | GCGACTTCTGTAAAGTTTATTTACAAGATAACAAAGAAACTCCCTTAAGCCAGCTGAAGCTTCGTACGCTG |
|  | Aro10 Del R | CAGAAAACGAACAATTGGTAGCAGTGTTTTATAATTGCGCCCACAAGTTTGGCCGCATAGGCCACTAGTGGATCTG |
|  | Aro10 F chk | GATATAAAACATATTTAACTGATCAACCCTC |
|  | Aro10 R chk | TACTAGCAACTGGACAAAGAACTCTG |
|  | cdc19-kanMX F | CTTCCACAATTTCGGCTCTATTG |
|  | cdc19-kanMX R | GAAGAATAGGACGGAGTAGC |
|  | ZWF1 del F | ATGAGTGAAGGCCCCGTCAAATTCGAAAAAAATACCGTCATATCTGTCTTCAGCTGAAGCTTCGTACGCTG |
|  | ZWF1 del R | CTAATTATCCTTCGTATCTTCTGGCTTAGTCACGGGCCAAGCGTAAGGGTGGCCGCATAGGCCACTAGT |
|  | pGREG-hphNT1 F | TACAAATGACAAGTTCTTGAAAACAAGAATCTTTTTATTGTCAGTACTGATTATTCCTTTGCCCTCGGACGA |
|  | pGREG-hphNT1 R | AGCTCAGGGGCATGATGTGAC |
|  | pGREG-hphNT1 chk F | AGTTATGTCACGCTTACATTCACGC |
|  | *pGREG-hphNT1 chk R | GACGACACCGTCAGTGCG |
| **Sequencing** | | |
|  | EF89.506_6OMT_frw | ATGACCATGATTACGCCAAGCG |
|  | EF96.4OMT_506_rev | CGATACTAACGCCGCCATCC |
|  | JF24 | AAGAAAGCGAAAGGAGCGGGG |
|  | pYES2 rev sequencing | CCTGGTATCTTTATAGTCCTGTCG |
|  | 181.seqFBA1p_f | CTCTCTTTCCATATCTAACAACTGAG |
|  | 182.seqFBA1p_r | CTCAGTTGTTAGATATGGAAAGAGAG |
|  | 183.seqGPD1p_f | CACACCACCAATACGTAAACGG |
|  | 184.seqGPD1p_r | CCGTTTACGTATTGGTGGTGTG |
|  | 185.seqHXT7p_f | GGCAACTGAAAGAATGAAAAGGAG |
|  | 186.seqHXT7p_r | CTCCTTTTCATTCTTTCAGTTGCC |
|  | 187.seqPDC1p_f | GACGGTGTCTTGATCTACTTGC |
|  | 188.seqPDC1p_r | GCAAGTAGATCAAGACACCGTC |
|  | 189.seqPGK1p_f | GGCTCACAGGTTTTGTAACAAGC |
|  | 190.seqPGK1p_r | GCTTGTTACAAAACCTGTGAGCC |
|  | 191.seqPYK1p_f | GACAGATTGGGAGATTTTCATAGTAG |
|  | 192.seqPYK1p_r | CTACTATGAAAATCTCCCAATCTGTC |
|  | 193.seqTEF1p_f | CGATGACCTCCCATTGATATTTAAG |
|  | 194.seqTEF1p_r | CTTAAATATCAATGGGAGGTCATCG |
|  | 195.seqTEF2p_f | AGTTGCTGACAGAAGCCTCAAG |
|  | 196.seqTEF2p_r | CTTGAGGCTTCTGTCAGCAACT |
|  | 197.seqTPIp_f | GTGGCATGTGAGATTCTCCG |
|  | 198.seqTPIp_r | CGGAGAATCTCACATGCCAC |
|  | 199.seqADH1t_f | GGTCAAGTCTCCAATCAAGGTTG |
|  | 200.seqADH1t_r | CAACCTTGATTGGAGACTTGACC |
|  | 201.seqADH2t_f | CGCGTTTGCTAGCACGAGTG |
|  | 201.seqADH2t_r | CACTCGTGCTAGCAAACGCG |
|  | 203.seqCYC1t_f | CCGAAAAGGAAGGAGTTAGACAAC |
|  | 204.seqCYC1t_r | GTTGTCTAACTCCTTCCTTTTCGG |
|  | 205.seqENO2t_f | CTGTCTATTGCATAATGCACTGGAAG |
|  | 206.seqENO2t_r | CTTCCAGTGCATTATGCAATAGACAG |
|  | 207.seqFBA1t_f | CAAAGTCATCCTAATCGATCTATCG |
|  | 208.seqFBA1t_r | CGATAGATCGATTAGGATGACTTTG |
|  | 209.seqPGI1t_f | CAGCGTCCAAGTAACTACATTATG |
|  | 210.seqPGI1t_r | CATAATGTAGTTACTTGGACGCTG |
|  | 211.seqTDH2t_f | GTAACATAACCTGAAGCATAACTGAC |
|  | 212.seqTDH2t_r | GTCAGTTATGCTTCAGGTTATGTTAC |
|  | 213.seqTPI1t_f | GCATTTAACAATTGAACACCTCTATATC |
|  | 214.seqTPI1t_r | GATATAGAGGTGTTCAATTGTTAAATGC |
|  | JF104.seqTDH3p_f | GAAACCAGTTCCCTGAAATTATTCC |
|  | JF105.seqTDH3p_r | GGGTTACAGCAGAATTAAAAGGC |
|  | seq.ARO7 F | GATTATACCATTAATTTCGAAAAGAGATGG |
|  | seq.ARO7 R | CCATCTCTTTTCGAAATTAATGGTATAATC |
|  | seq.TDH3p F | CAGTTCATAGGTCCATTCTCTTAGC |
|  | seq.TDH3p R | GCTAAGAGAATGGACCTATGAACTG |
|  | seq.TyrC F | AGCACAGGCTATCGCCTATATC |
|  | seq.TyrC R | GATATAGGCGATAGCCTGTGCT |
|  | seq.ARO1.1 F | CATACTACCAGCAATTAGTCCTGG |
|  | seq.ARO1.1 R | CCAGGACTAATTGCTGGTAGTATG |
|  | seq.ARO1.2 F | GAACGTGAATCCAGTCTAAGAAACC |
|  | seq.ARO1.2 R | GGTTTCTTAGACTGGATTCACGTTC |
|  | seq.ARO1.3 F | CATGGTGGTTCCACATTGTCAG |
|  | seq.ARO1.3 R | CTGACAATGTGGAACCACCATG |
|  | seq.ARO1.4 F | CTCAAACGGCAACTTCAACTACTG |
|  | seq.ARO1.4 R | CAGTAGTTGAAGTTGCCGTTTGAG |
|  | seq.ARO1.5 F | GTTGACCTAGACGAGCTGTTTG |
|  | seq.ARO1.5 R | CAAACAGCTCGTCTAGGTCAAC |
|  | seq.ARO1.6 F | CAGTATTCCTATCATTTTTACTGTGCG |
|  | seq.ARO1.6 R | CGCACAGTAAAAATGATAGGAATACTG |
|  | seq.ARO1.7 F | CTGAATCCGCACAATTGGTGAAAG |
|  | seq.ARO1.7 R | CTTTCACCAATTGTGCGGATTCAG |
|  | seq.ARO1.8 F | GTAAGCTGGAGAGATTCCTTGTG |
|  | seq.ARO1.8 R | CACAAGGAATCTCTCCAGCTTAC |
|  | S2Aro4rev | TTGCAAACCCTTGTTGATGTTGAA |
|  | S3Aro4for | TTCAACATCAACAAGGGTTTGCAA |
|  | S3Aro4rev | GGTTACCTTCGTTGATGTTTGATTC |
|  | S4Aro4for | GAATCAAACATCAACGAAGGTAACC |
|  | TYR1_seq_1F | ACAGTACCCAGAATCTTTTG |
|  | TYR1_seq_2F | TTTGTTCGACGCCATTATTC |
|  | TYR1_seq_1R | CCAAGTTGAAACCACGAATC |
|  | TYR1_seq_2R | GAATGCACGGTAATGATGTC |
